# Soluble Nanomedicine Against Soluble Neurosensitizers to Effectively Relieve Osteoarthritis Pain and Joint Damage

**DOI:** 10.1002/exp2.70233

**Published:** 2026-10-08

**Authors:** Junyan Liu, Feixiang Ding, Jiayi Li, Min Liu, Lingyu Su, Zixi Wang, Congying Pi, Shilong Jiang, Zhaokui Zeng, Changjun Li, Chao Zeng, Guanghua Lei, Kelong Ai

**Affiliations:** ^1^ Department of Orthopaedics The Third Xiangya Hospital Central South University Changsha China; ^2^ Department of Orthopaedics Xiangya Hospital Central South University Changsha China; ^3^ Xiangya School of Pharmaceutical Sciences Central South University Changsha China; ^4^ Department of Pharmacology and Chemical Biology College of Basic Medical Sciences Shanghai Jiao Tong University School of Medicine Shanghai China; ^5^ Department of Pharmacy Xiangya Hospital Central South University Changsha China; ^6^ Key Laboratory of Aging‐Related Bone and Joint Diseases Prevention and Treatment Ministry of Education Xiangya Hospital Central South University Changsha China; ^7^ Department of Endocrinology Endocrinology Research Center Xiangya Hospital of Central South University Changsha China; ^8^ Hunan Key Laboratory of Joint Degeneration and Injury Changsha China; ^9^ National Clinical Research Center for Geriatric Disorders Xiangya Hospital Central South University Changsha China

**Keywords:** joint pain, neurosensitizer, osteoarthritis, sensory perception, synovial inflammation

## Abstract

Targeting soluble neurosensitizers in the osteoarthritis (OA) joint cavity is a key strategy for alleviating OA pain. Soluble neurosensitizers are ubiquitous in the joint cavity and cause uncontrollable growth and sensitization of nociceptor axons therein. However, eliminating these soluble nerve growth factors (NGFs) remains a considerable challenge due to the diversity of neurosensitizers and the complexity of OA pathology. This study introduces an innovative soluble nanodrug (LAC)‐based strategy to eliminate three key neurosensitizers (H^+^, NGF, and netrin‐1) to simultaneously relieve OA‐associated pain and joint damage. In two animal model experiments, LAC significantly reduced pain and alleviated OA progression by reducing synovial inflammation and inhibiting cartilage and subchondral bone damage. This strategy of using soluble nanomedicine to counter soluble neurosensitizers not only provides inspiration for eliminating pain, but also sheds light on possible solutions for advancing OA treatment strategies.

## Graphical abstract

Osteoarthritis pain arises not only from joint damage but also from inflammation, abnormal bone changes, and excessive nerve activation inside the joint. In this study, we developed an injectable nanomedicine, LAC, that responds to the acidic environment of osteoarthritic joints to locally release two approved drugs, reducing inflammation, limiting bone damage, neutralizing acidity, and preventing pain‐related nerve overgrowth. This targeted, multi‐action approach may offer a safer and more effective way to relieve osteoarthritis pain while potentially slowing disease progression.

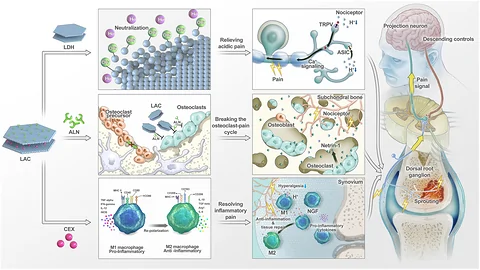

## 1 Introduction

Osteoarthritis (OA), a degenerative joint disease caused by the destruction of cartilage and subchondral bone, is a leading cause of pain and disability worldwide [1, 2]. The prevalence of OA has doubled in recent decades, with estimates suggesting that more than 500 million people worldwide suffer from OA, and more than one in five adults over 40 years of age has knee OA [3, 4]. Currently, OA poses major public health challenges [5]. Pain is not only a hallmark symptom of OA, but also the main motivation for patients with OA to seek medical treatment and the main cause of functional limitation and decreased quality of life [6, 7]. To date, clinical approaches for managing OA pain, including physical therapy, oral analgesics, nonsteroidal anti‐inflammatory drugs (NSAIDs), and intra‐articular injections (corticosteroids or hyaluronic acid), provide only limited and short‐term symptom relief, and none can effectively prevent OA progression [8, 9]. Physical therapy and lifestyle modifications primarily reduce mechanical stress but have modest efficacy for moderate or severe pain. Pharmacologic treatments, such as acetaminophen, NSAIDs, antidepressants, and humanized nerve growth factor (NGF) monoclonal antibodies, are restricted by low efficacy or significant adverse effects [10, 11]. Intra‐articular injections can offer temporary improvement but do not modulate the molecular drivers of chronic OA pain and may even accelerate joint degeneration in susceptible individuals. These limitations highlight an unmet need for therapeutic strategies that can both suppress OA pain and target the underlying biochemical drivers of nociceptor sensitization. Therefore, effective pain‐suppressing drugs are urgently needed for the treatment of OA.

The mechanisms involved in OA pain are highly complex and not always closely related to joint damage [12, 13]. Up to 40% of patients with OA who have joint damage do not experience pain, whereas patients whose cartilage damage is unusually mild or even radiographically undetectable may experience considerable debilitating pain [14, 15]. This puzzling phenomenon is related to soluble neurosensitizers in OA joints [16, 17]. The cell bodies of specialized pain‐sensing neurons, called nociceptors, are located within the dorsal root ganglia (DRG) [18, 19]. The nociceptors extend one axon into the joint and another into the dorsal horn of the spinal cord. Pain signals are transmitted from the DRGs to the dorsal horn and then relayed to the brain, where a pain sensation is produced [20, 21]. Specialized nociceptor axons are abundant in the synovium and subchondral bones of joints [22, 23]. As OA progresses, nociceptors are sensitized by specific soluble nerve sensitizers in OA joints, and the pain escalates to severe or chronic pain, seriously reducing the quality of life of patients [3, 24]. Specifically, synovial inflammation leads to acidification of the microenvironment, which stimulates H^+^‐sensitive channels of sensory nerve fibers to produce severe pain [25, 26]. Moreover, M1‐polarized macrophages in the synovium and osteoclasts in the subchondral bone drive gliosis and abnormal synapse formation by NGF and netrin‐1, increasing neuronal excitability and causing severe allodynia [11, 27, 28]. Therefore, therapeutic strategies aimed at eliminating these soluble neurosensitizers in OA joints may provide a potential treatment for OA pain.

Layered double hydroxides (LDHs) are a class of two‐dimensional nanomaterials that have been extensively explored in biomedical applications, including drug delivery, cancer therapy, and tissue engineering, owing to their high specific surface area, tunable composition, and excellent biocompatibility and degradability [29, 30, 31]. In orthopedic‐related research, LDHs have been predominantly investigated for their osteogenic and bone‐regenerative properties, either as drug carriers, scaffold additives, or bioactive components that release metal ions (e.g., Mg^2^^+^ and Fe^3^^+^) to promote osteoblast differentiation, mineralization, and osseointegration [32, 33, 34]. Accordingly, most LDH‐based strategies in musculoskeletal applications are designed to enhance bone repair or structural integration. In contrast, the potential role of LDHs in modulating the joint microenvironment associated with pain has received comparatively little attention.

Here, we propose LAC, a nanocomposite specifically engineered to simultaneously eliminate the major soluble neurosensitizers that drive OA pain. (Figure 1A). LAC is composed of three key components: Mg/Al‐LDH, celecoxib (CEX), and alendronate sodium (ALN) [35, 36], which can effectively eliminate soluble neurosensitizers to relieve OA pain through synergistic effects. Specifically, the pain factor, H^+^, in OA can be neutralized and eliminated by LDH. During this process, LDH is dissolved, and CEX and ALN are gradually released. Among them, CEX can induce transformation of the macrophage phenotype to the M2 type, which not only reduces synovial inflammation in OA but also significantly reduces secretion of the neurotrophic factor, NGF. ALN can inhibit osteoclast activity, which markedly reduces the destruction of subchondral bone and also reduces the release of the neurotrophic factor, netrin‐1. Ultimately, the three key soluble neurosensitizers were significantly reduced, thereby preventing the formation of hyperalgesia and allodynia. Moreover, LAC effectively eliminated synovial inflammation, cartilage loss, and subchondral bone destruction, and its therapeutic effects were encouraging. Overall, this study provides a paradigm for the efficient treatment of OA by simultaneously relieving pain symptoms and preventing OA progression.

**FIGURE 1:**
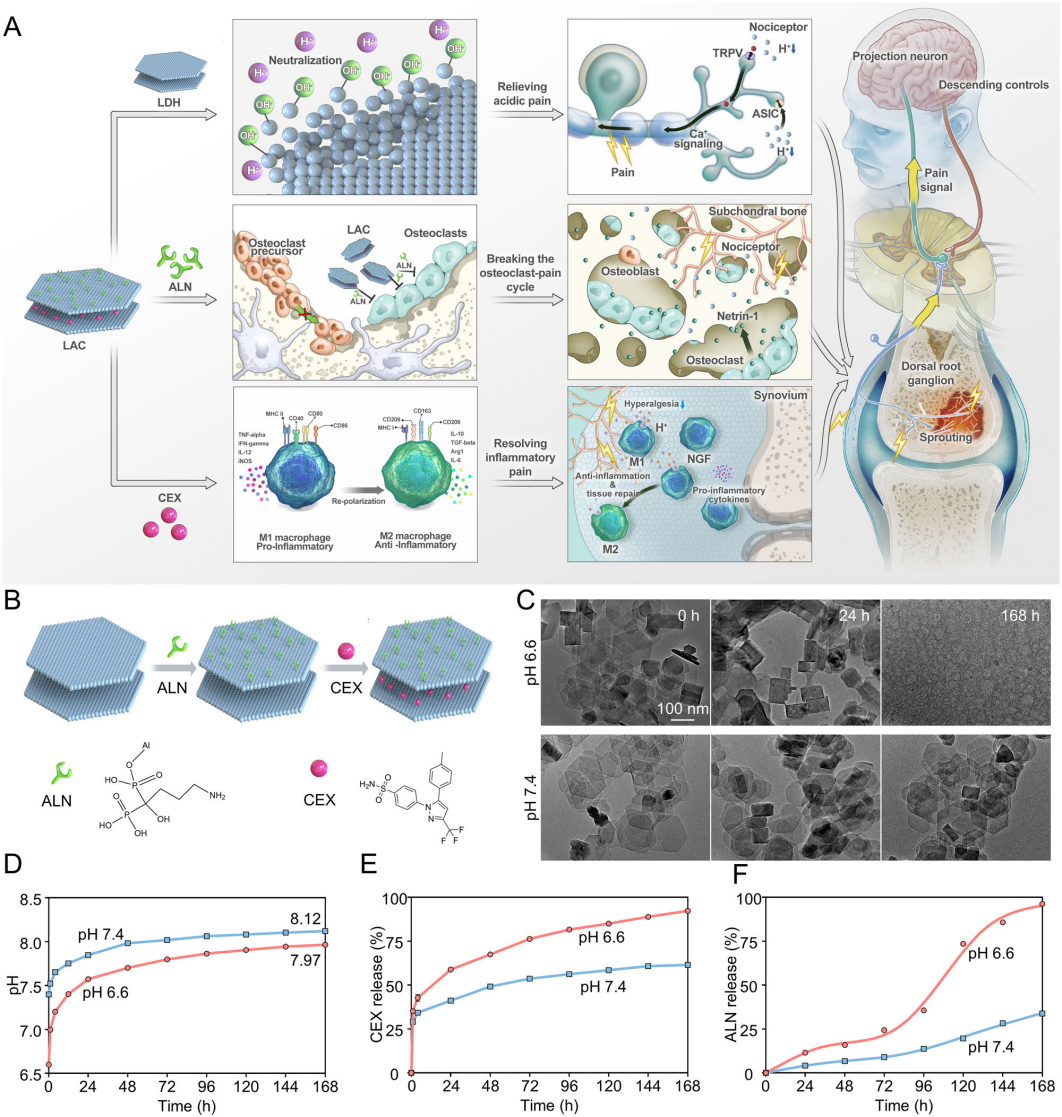
Schematic illustration of the synthetic procedure and therapeutic mechanism of LAC on OA and the corresponding characterization. (A) Therapeutic mechanism of LAC on OA. (B) Synthetic procedure of LAC. (C) TEM images of LAC in pH 6.6 and pH 7.4 buffer solution at the time of 0, 24, and 168 h. (D) pH change of LAC solution with an initial pH of 6.6 and 7.4 within 7 days. (E,F) Cumulative release curve of CEX (E) and ALN (F) from LAC in pH 6.6 and pH 7.4 PBS within 7 days. *n* = 3, mean ± SD.

## 2 Results and Discussion

### 2.1 Synthesis and Characterization of LAC

LAC was prepared by a three‐step process: the LDH support was first synthesized by a hydrothermal method; then, ALN was modified on the LDH surface via Al─O─P coordination bonds; and finally, CEX was loaded into the voids of the layered structure of LDH (Figure 1B). Transmission electron microscopy (TEM) revealed that LAC had a hexagonal sheet structure similar to that of LDH, with a particle size of approximately 190 nm (Figure 1C), indicating that the modification of ALN and loading of CEX did not affect the crystal structure of LAC. When the electronegative CEX was loaded into the interlayer space of the positively charged LDH, an increase in the hydrodynamic diameter and a decrease in the surface potential occurred. The hydrodynamic diameter of LAC (304.5 nm) was slightly larger than that of LDH (198.2 nm) (Figure S1A). Moreover, zeta potential measurements showed that the surface potential of LAC (−11.8 mV) was significantly lower than that of LDH (16.8 mV) (Figure S1B). In addition to harboring the same O, C, Mg, and Al as those of LDH, LAC contained F and S derived from CEX and N and P derived from ALN, as determined through x‐ray photoelectron spectroscopy (Figure S2). Through assessing the fine peak of C1s, LAC was found to contain the C─O/C─N peak (285.7 eV) derived from CEX and ALN (Figure S3). LAC also contained the F1s (685.2 eV) and S2p (167.9 eV) peaks attributed to the C─F and sulfonamide of CEX (Figure S4). The N1s peak of LAC consisted of two peaks located at 401.4 and 399.5 eV, which are attributed to the amino groups of ALN and CEX and the pyrrolic N of CEX, respectively (Figure S6). Similarly, the P2p peak at 133.5 eV in LAC was divided into p1/2 (134.2 eV) and p3/2 (132.2 eV) peaks, which could be attributed to the phosphate bonds in ALN (Figure S6). Notably, the O1s (Figure S5), Mg2p (Figure S7), and Al2p (Figure S8) belonging to LDH did not significantly change after ALN and CEX loading, indicating that this did not affect the presence of Mg^2+^ and Al^3+^ in LDH. Through x‐ray diffraction, LDH and LAC were observed to have crystal diffraction peaks with consistent positions. Owing to the interlayer enrichment of CEX in LDH, the crystallinity of LDH was reduced, and the peak intensities of LAC at 11.27° (003) and 22.78° (006) were significantly reduced (Figure S9). The loading rate of CEX in LAC was 5.73%  ± 1.43%, according to UV–vis spectroscopy (Figure S10). Based on the *o*‐phthalaldehyde (OPA) assay, the ALN loading rate in LAC was 15.48% ±  0.07% (Figure S11). Overall, these results confirm that LAC was successfully prepared.

Synovial inflammation is closely associated with the acidic microenvironment in OA [37, 38]. The pH values of normal and arthritic synovial fluid are 7.4 and 6.6, respectively [39]. Thus, LAC was dispersed in a pH 6.6 buffer to evaluate its degradation in acidic synovial fluid typical of OA. As shown in Figure 1C, H ^+^ slightly altered the hexagonal crystal form of LAC within 24 h, as observed through TEM, and was almost completely degraded after 168 h. In contrast, at pH 7.4, the morphological changes in LAC were almost negligible. Correspondingly, LAC reacted rapidly with H^+^ at pH 6.6, causing the pH to rise to approximately 7 within 4 h, whereafter the reaction rate decreased significantly, reaching a stable pH of 7.97 within 168 h (Figure 1D). At pH 7.4, LAC caused a pH change of less than 1.0 within 168 h. Notably, the final stabilized pH of 7.97 resided within a physiologically well‐tolerated range and was postulated to exert a protective effect by ameliorating the pathological acidosis prevalent in the osteoarthritic joint microenvironment. The release of CEX, ALN, and Mg^2+^ in LAC was further explored at pH 6.6 and 7.4. As shown in Figure 1E, the amount of released CEX increased from 61.60% at pH 7.4 to 92.34% at pH 6.6 within 7 days. Similarly, the amount of released ALN increased from 38.81% at pH 7.4 to 96.28% at pH 6.6 (Figure 1F). In parallel, the Mg^2+^ release profile further confirmed the acid‐triggered dissolution of the LDH framework (Figure S12). These results demonstrate that LAC can remain intact in a neutral pH environment while effectively targeting the acidic OA environment to rapidly collapse, leading to in situ H^+^ clearance and the release of CEX and ALN, thereby minimizing the off‐target effects of treatment.

### 2.2 Long‐Term Retention to Osteoarthritic Joints and the Effective Relief of Pain by LAC

Therapeutic drugs for OA need to be efficiently concentrated at the lesion site and have a long retention time because OA is a chronic disease [40, 41]. The monosodium iodoacetate (MIA) model was induced via intra‐articular injection of sodium iodoacetate [42, 43]. LAC was first labeled with the fluorescent dye, Cy7 (LAC─Cy7) (Figure S13), whereafter it was injected into the OA joint cavity to track its distribution in the MIA model using a small animal in vivo imaging system (Figure 2A). Small‐molecule drugs can be easily excreted through the circulatory system via diffusion after they are injected into the joint cavity [44]. Fortunately, as a nanomedicine, LAC has physicochemical properties that are different from those of small‐molecule drugs. After joint cavity injection, LAC remained in the joint instead of being excreted through diffusion. As shown in Figure 2B, Cy7‐LAC was enriched in OA joints, and 5 days after injection, the fluorescence intensity in the joint cavity dropped to approximately half of the initial level. Although the fluorescence signal weakened, a relatively obvious fluorescence signal could still be observed in the joint cavity even 7 days after injection until 14 days after injection, demonstrating the long‐term retention of LAC in the joint cavity (Figure 2C). We further isolated and analyzed the synovial tissue of OA joints from the MIA model following intra‐articular injection of Cy7‐LAC. Unlike the gradual decrease in fluorescence intensity observed from Day 1 in the in vivo imaging analysis, ex vivo analysis revealed an increase in fluorescence intensity between Days 1 and 3. This discrepancy is likely attributable to the time required for Cy7‐LAC to penetrate deeply into the synovial tissue (Figure 2D and Figure S14). Moreover, TEM images demonstrated that LAC was predominantly localized within macrophages in the synovial tissue, and TEM‐energy‐dispersive x‐ray spectroscopy further confirmed a uniform intracellular distribution of Mg and Al within synovial cells (Figure S15). Consistently, immunofluorescence staining of synovial tissues collected 5 days post‐injection using the macrophage marker F4/80 showed pronounced co‐localization of Cy7‐LAC with synovial macrophages (Figure S16). Collectively, these findings indicate that LAC delivered via intra‐articular injection can persist in OA joints for an extended period and is efficiently internalized by synovial macrophages, thereby providing a mechanistic basis for its therapeutic effects in alleviating OA‐associated pain and synovial inflammation.

**FIGURE 2:**
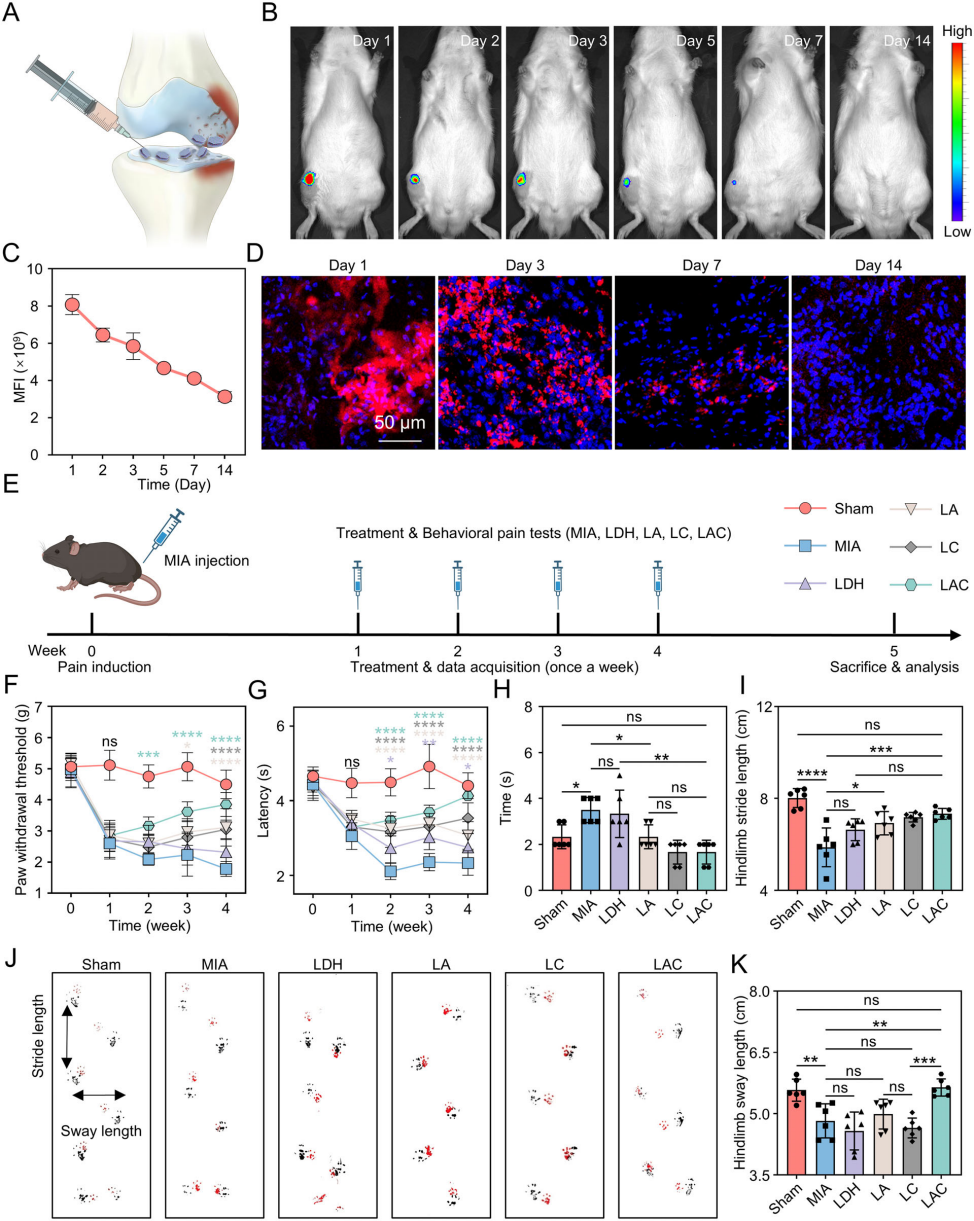
Characterization of LAC. (A)Schematic illustration of intra‐articular injection of Cy7‐labeled LAC. (B) Representative fluorescence images of MIA rats after intra‐articular injection of Cy7‐labeled LAC at different time points. (C) Mean fluorescence intensity (MFI) curve of the knee joint at different time points. (D) Representative fluorescence images of the fluorescence in the knee joint after injection of Cy7‐labeled LAC. (E) The establishment and therapeutic regimen of the MIA mouse model. (F) The von Frey test for mechanical hypersensitivity. (G) The hot plate test for thermal hypersensitivity. (H) The time used to cross the tunnel. (I) Histogram represents differences in hindlimb stride length. (J) Representative footprints of mice with different treatments. Red prints for the fore paw, and black prints for the hind paw. (K) Histogram represents differences in hindlimb sway length. n = 3 in C, and *n* = 6 in F‐–K, mean ± SD. The significant difference is determined by two‐way ANOVA with Tukey's multiple comparison test in F and G, and by one‐way ANOVA with Tukey's multiple comparison test in H–K. *p < 0.05, **p < 0.01, ***p < 0.001, ****p < 0.0001, ns stands for no significant difference.

Building on the sustained intra‐articular retention of LAC and its preferential uptake by synovial macrophages, we next investigated its therapeutic efficacy in alleviating OA‐associated pain in MIA‐induced mice. MIA mice were treated with varying concentrations of LAC via intra‐articular injection for four consecutive weeks, and pain‐related behaviors were evaluated weekly using weight‐bearing and von Frey tests (Figure S17). As OA progressed, MIA mice exhibited a persistent reduction in ipsilateral hindlimb weight‐bearing and paw withdrawal thresholds, indicative of increasing pain sensitivity. In contrast, mice receiving LAC treatment displayed dose‐dependent attenuation of pain behaviors, with distinct therapeutic efficacies observed among different concentrations. Notably, although the analgesic effects varied across doses, the 125 µg kg^−1^ LAC treatment group achieved a pronounced therapeutic benefit at a relatively low dose. By the end of the treatment period, both bilateral hindlimb weight‐bearing and paw withdrawal thresholds in this group were restored to levels comparable to those of the Sham group, with no statistically significant differences observed. Therefore, 125 µg kg^−1^ was determined to be the optimal therapeutic dose of LAC for subsequent experiments. Next, the analgesic efficacies of LDH, LC (LDH loaded with CEX), LA (LDH loaded with ALN), and LAC were evaluated at equivalent doses in the MIA‐induced OA model (Figure 2E). As shown in Figure 2F, von Frey testing revealed progressive mechanical hypersensitivity in MIA mice, with paw withdrawal thresholds declining from an initial value of 4.98 ± 0.37 to 1.78 ± 0.24 g, compared with a stable threshold of 4.50 ± 0.46 g in the Sham group, indicating continuous OA‐associated mechanical pain. Treatment with LA, LC, and LAC significantly ameliorated mechanical hypersensitivity relative to the MIA group. Notably, mice treated with LAC exhibited the most pronounced recovery, with paw withdrawal thresholds increasing to 3.85 ± 0.38 g, approaching levels comparable to those of the Sham group. Consistent results were obtained in the hot plate test (Figure 2G). As OA progressed, the latency time in MIA mice decreased from 4.43 ± 0.36 to 2.33 ± 0.33 s, whereas treatment with LA, LC, and LAC significantly prolonged response latency (LA: *p* < 0.01; LC: *p* < 0.0001; LAC: *p* < 0.0001), with LAC again demonstrating the strongest analgesic effect. Bipedal weight‐bearing analysis further corroborated these findings (Figure S18). Moreover, footprint analysis revealed that OA mice displayed pronounced gait asymmetry, prolonged tunnel‐crossing time, reduced forelimb and hindlimb stride length, and narrowed sway length compared with Sham mice (Figure 2H–K and Figure S19). While administration of LA and LC resulted in partial improvements in these motor and gait parameters, LAC treatment produced significantly greater restoration across all indices, indicative of a marked attenuation of spontaneous pain‐related behaviors. Overall, these results support that while delivery of a single therapeutic component (LA and LC) partially alleviates OA pain, co‐loading of ALN and CEX within LAC yields enhanced and more comprehensive pain relief.

### 2.3 Transcriptomic Analysis of the Therapeutic Effect of LAC

Given the preferential accumulation of LAC in synovial macrophages and its pronounced analgesic effects, we next performed transcriptomic sequencing to elucidate the molecular mechanisms underlying LAC‐mediated modulation of synovial macrophage function. Synovial tissues were harvested from mice in the Sham, MIA, and LAC treatment groups, and total RNA was extracted for high‐throughput sequencing. Venn diagram analysis revealed distinct transcriptional profiles among the MIA/Sham, LAC/Sham, and LAC/MIA comparisons. Notably, the MIA/Sham comparison exhibited the largest number of uniquely expressed genes (430), whereas the transcriptomic profile of the LAC‐treated group more closely resembled that of the Sham group, indicating partial normalization of OA‐associated gene expression following LAC treatment (Figure S20A). Volcano plot analysis further showed that, relative to the MIA group, the LAC group exhibited 1557 differentially expressed genes (DEGs), including 384 upregulated and 1173 downregulated genes (Figure S20B). To identify pathways associated with the therapeutic effects of LAC, Kyoto Encyclopedia of Genes and Genomes (KEGG) pathway enrichment analysis was performed on the DEGs. Compared with the MIA group, LAC treatment was associated with marked downregulation of pathways related to cytokine–cytokine receptor interaction, neuroactive ligand–receptor interaction, and axon guidance (Figure 3A). Moreover, consistently, Gene Ontology (GO) enrichment analysis and GO chord plot indicated that LAC treatment positively regulated ion transmembrane transport while suppressing biological processes associated with inflammatory responses, cellular responses to tumor necrosis factor, and neuron projection development (Figure 3B and Figure S21). Gene Set Enrichment Analysis (GSEA) further corroborated these findings, demonstrating significant downregulation of cytokine‐mediated signaling (NES = −1.78), response to mechanical stimulus (NES = −1.51), sensory perception of pain (NES = −1.53), T cell receptor signaling (NES = −1.65), cation transmembrane transport (NES = −1.52), and axon guidance (NES = −1.47) in the LAC‐treated group (Figure 3C–H). Selected genes enriched in these pathways were further validated by RT–qPCR. As shown in Figure 3I–L, LAC treatment markedly reduced the mRNA expression of *Cd24* (a marker of inflammatory macrophage activation and synovial inflammation), *Chi3l1* (associated with OA‐related inflammation and tissue remodeling), *Ccl19* (a chemokine involved in immune cell recruitment and neuroinflammation), and *Mmp9* (a matrix‐degrading enzyme implicated in cartilage destruction, synovial inflammation, and subchondral bone remodeling), with reductions of 256.43‐fold, 1.46‐fold, 2.41‐fold, and 1.80‐fold, respectively, compared with MIA mice. However, there were also genes like *Ccl9*, *Klf2*, and *Kcna5* that showed negligible differences between OA mice and mice with different treatments (Figure S22). Together, transcriptomic profiling revealed that LAC treatment was associated with coordinated changes in genes linked to macrophage activation, inflammatory signaling, axonogenesis, and neuronal excitability. Notably, pathways related to cytokine–chemokine signaling and ion homeostasis were enriched among the DEGs. While these changes suggest a potential link between LAC treatment and reduced neuroinflammatory and nociceptive signaling, the functional consequences of these transcriptomic alterations remain to be determined.

**FIGURE 3:**
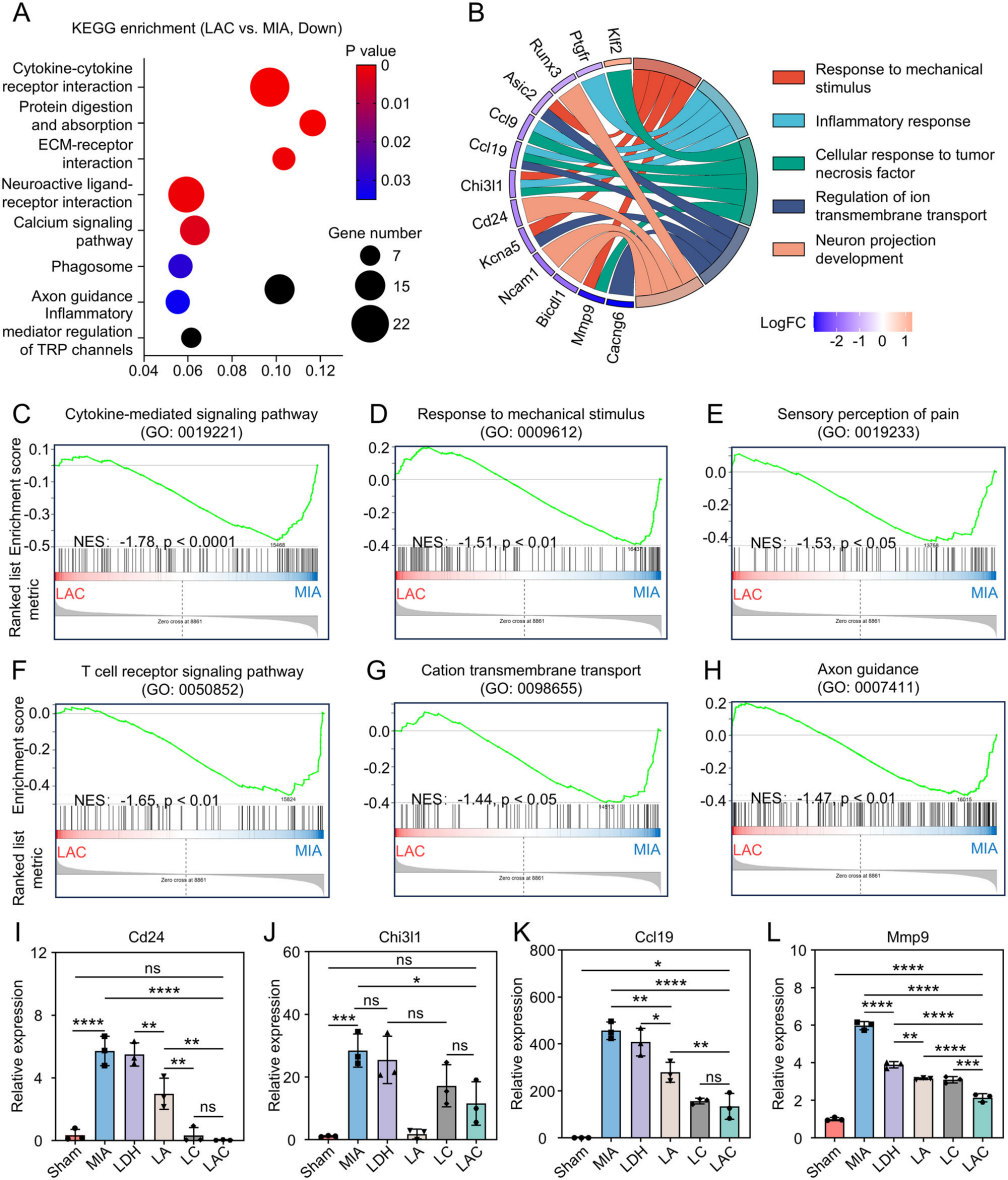
Transcriptomic analysis. (A) Differential gene Kyoto Encyclopedia of Genes and Genomes (KEGG) enrichment pathway for LAC when compared to the MIA group. (B) Gene Ontology (GO) chord plot showing the relationship between DEGs and their associated GO terms. (C–H) Gene Set Enrichment Analysis (GSEA) graphs of the indicated signaling pathway in LAC compared with those in the MIA group. (I–L) Relative expression of Cd24 (I), Chi3l1 (J), Ccl19 (K), and Mmp9 (L) in synovial tissues of mice with different treatments. Data are presented as mean ± SD, *n* = 3. Significant differences were determined via one‐way ANOVA analysis with Tukey's multiple comparison test. *p < 0.05, **p < 0.01, ***p < 0.001, ****p < 0.0001, ns stands for no significant difference.

### 2.4 M1 Macrophage Repolarization and Osteoclast Deactivation by LAC

Based on the transcriptomic evidence indicating reduced synovial inflammation, we next investigated whether LAC could modulate macrophage phenotypic polarization, particularly the transition from pro‐inflammatory M1 macrophages to an anti‐inflammatory M2 phenotype. RAW264.7 cells were first polarized toward the M1 phenotype by stimulation with lipopolysaccharide (LPS) and interferon‐γ (IFN‐γ) (LPS group) before subsequent treatments. Consistent with an inflammatory microenvironment, we found that LPS stimulation induced marked acidification of the culture medium (pH ∼6.4), closely resembling pathological conditions in OA joints (Figure S23).

We initially examined the cellular uptake of Cy7‐labeled LAC by M1 macrophages using flow cytometry (FCM). Time‐course analysis revealed a progressive increase in intracellular fluorescence intensity, reaching a plateau at approximately 12 h, indicative of efficient endocytic uptake of Cy7‐LAC (Figure S24). M1‐polarized RAW264.7 cells were then treated with increasing concentrations of LAC to evaluate its effects on macrophage polarization. Western blot analysis identified 20 µg mL^−^^1^ as the minimum effective concentration capable of significantly downregulating cyclooxygenase‐2 (COX‐2) and inducible nitric oxide synthase (iNOS), while concomitantly upregulating the M2 marker CD206 (Figure S25).

Using this concentration, we further analyzed the expression of M1/M2‐associated surface markers and key transcription factors. As shown in Figure 4A, the expression of COX‐2, iNOS, and interferon regulatory factor 5 (IRF5) was markedly increased in the LPS group compared with the Control (Ctrl) group. In contrast, LAC treatment significantly reduced the expression of COX‐2, IRF5, and iNOS by 42.6%, 48.0%, and 60.7%, respectively, relative to the LPS group. Meanwhile, the expression of CD206 and phosphorylation of signal transducer and activator of transcription 6 (STAT6), a key transcription factor associated with M2 polarization, were increased by 80.8% and 83.5%, respectively. Interestingly, CEX treatment produced effects comparable to those of LAC, whereas LDH and ALN treatment resulted in negligible changes in the expression of these markers and transcription factors compared with the LPS group (Figure S26). Consistent trends were also observed in THP‐1‐derived macrophages, in which CEX and LAC reduced iNOS expression by 19.4% and 40.3%, respectively, and increased CD206 expression by 45.9% and 85.2%, respectively, compared with the LPS group (Figure S27).

**FIGURE 4:**
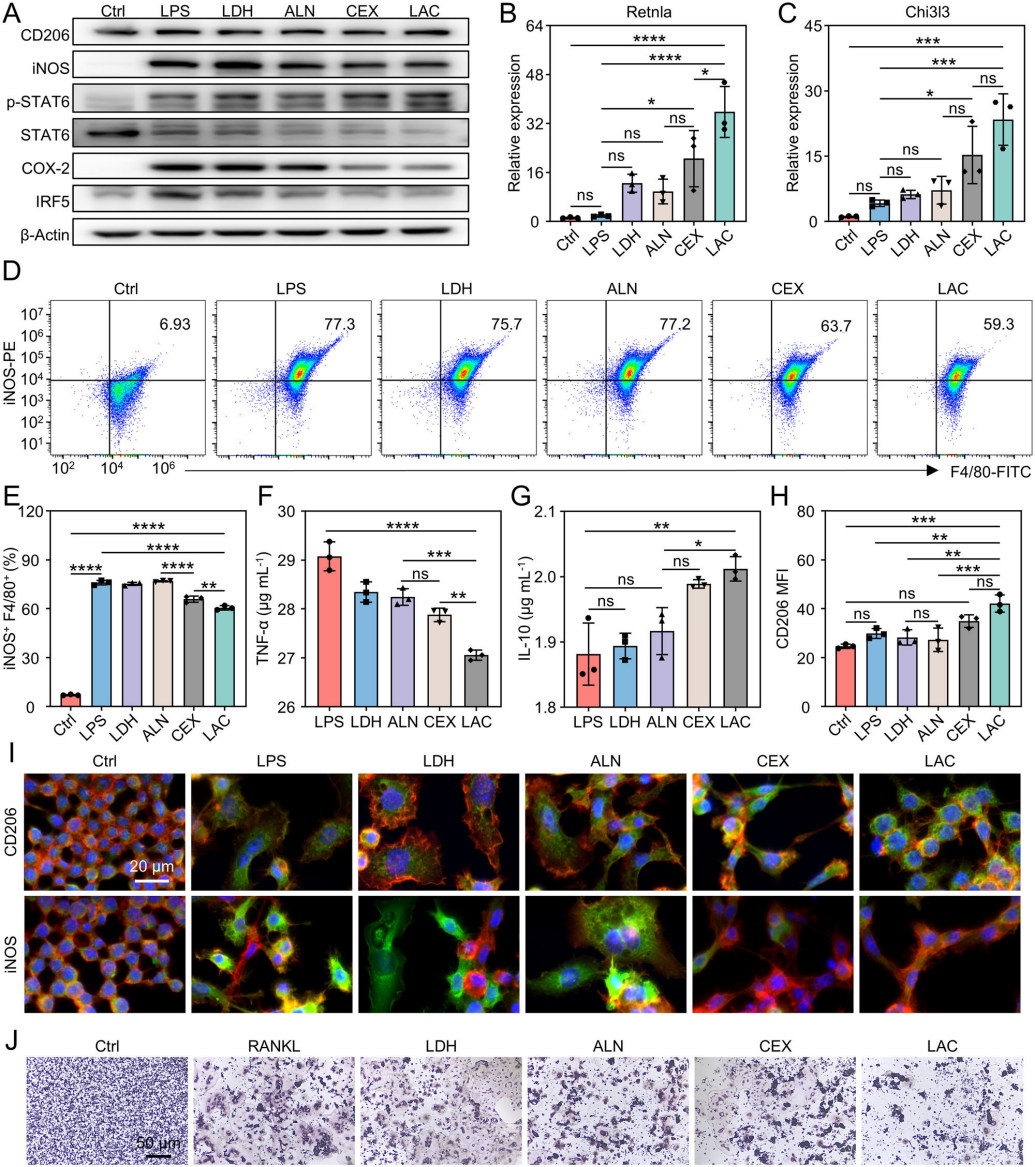
Reprogramming of M1 macrophage repolarization by LAC. (A) Representative Western blots of the expression of surface markers and the phosphorylation of transcription factors in the indicated treatments. (B) Relative expression of Retnla (B) and Chi3l3 (C) in cells with different treatments. (D,E) FCM profiles (D) and corresponding quantification of the proportion of M1 macrophages in the indicated groups. (F,G) Enzyme‐linked immunosorbent assay (ELISA)‐based measurements of TNF‐α (F) and IL‐10 (G). (H) Quantification of fluorescence intensity of CD206 in the indicated groups. (I) Immunofluorescence imaging of macrophages stained with DAPI (blue), phalloidin‐ Alexa Fluor 555 (red), together with CD206 or iNOS (green). (J) TRAP staining images of the TRAP‐positive multinucleated cells (MNCs) in the indicated group. Data are presented as mean ± SD, *n* = 3. Significant differences were determined via one‐way ANOVA analysis with Tukey's multiple comparison test. *p < 0.05, **p < 0.01, ***p < 0.001, ****p < 0.0001, ns stands for no significant difference.

To further validate M2 polarization, the expression of M2‐associated genes *Chi3l3* and *Retnla* was assessed by RT–qPCR. As shown in Figure 4B,C, *Retnla* expression in LAC‐treated cells was increased by 20.0‐fold relative to the LPS group, while *Chi3l3* expression was upregulated by 4.6‐fold. In contrast, LDH and ALN exhibit negligible M2 repolarization effect. Flow cytometric analysis further demonstrated that LAC treatment significantly altered macrophage subtype proportions. Compared with the LPS group, the percentage of F4/80^+^ iNOS^+^ macrophages decreased from 76.0% to 60.6%, whereas the proportion of F4/80^+^ CD206^+^ macrophages increased from 5.9% to 9.5% (Figure 4D,E and Figure S28). In parallel, immunofluorescence staining revealed distinct morphological changes: macrophages in the LPS, LDH, and ALN groups displayed a flattened and widely spread morphology characteristic of pro‐inflammatory M1‐like cells, whereas cells treated with CEX or LAC exhibited an elongated, spindle‐like morphology with extended cellular protrusions, a phenotype commonly associated with anti‐inflammatory M2‐like macrophages. Semi‐quantitative fluorescence analysis confirmed significant downregulation of iNOS and upregulation of CD206 in the LAC‐treated group compared with the LPS group (Figure 4H,I and Figure S29). In addition, analysis of macrophage‐secreted cytokines showed that LAC treatment reduced the level of the pro‐inflammatory cytokine TNF‐α by approximately 6.9% relative to the LPS group, while the concentration of the anti‐inflammatory cytokine IL‐10 increased by 7.0% (Figure 4F,G). Finally, to clarify whether Mg^2^^+^ released from the LDH carrier contributed to the observed immunomodulatory effects, prolonged incubation of RAW264.7 cells with LDH alone for up to 7 days did not induce detectable changes in M1/M2 marker expression, with COX‐2 and iNOS remaining undetectable throughout the incubation period (Figure S30). Collectively, these findings demonstrate that LAC effectively promotes macrophage polarization from a pro‐inflammatory M1 state toward an anti‐inflammatory M2 phenotype, an effect predominantly driven by the CEX component rather than LDH‐derived Mg^2^^+^, thereby providing a mechanistic basis for its anti‐inflammatory and analgesic effects observed in vivo.

In addition to its effects on macrophage polarization, we further investigated whether LAC could modulate receptor activator of nuclear factor κB ligand (RANKL)‐induced osteoclastogenesis and osteoclast activity. During OA progression, osteoclasts within the subchondral bone have been reported to secrete axon guidance factors, such as netrin‐1, which promote sensory nerve ingrowth into subchondral bone, enhance excitability of spinal dorsal horn neurons, and thereby exacerbate joint pain [28, 45]. To assess this effect, osteoclasts were continuously induced with 50 ng mL^−^^1^ RANKL and treated with different formulations, followed by Western blot analysis of netrin‐1 expression. As shown in Figure S31, compared with the RANKL‐induced group, netrin‐1 expression was reduced by approximately 30.3% and 37.8% in the ALN‐ and LAC‐treated groups, respectively, whereas no significant changes were observed in the LDH or CEX treatment groups. Consistently, tartrate‐resistant acid phosphatase (TRAP) staining revealed a marked reduction in osteoclast formation following ALN and LAC treatment. Specifically, the number of TRAP‐positive multinucleated osteoclasts in the LAC groups was approximately 3.27‐fold lower than that in the RANKL group (Figure 4J and Figure S32). Collectively, these results demonstrate that LAC effectively suppresses osteoclastogenesis and attenuates osteoclast‐derived netrin‐1 secretion, an effect that can be primarily attributed to the ALN component of LAC. This dual regulation of inflammatory macrophages and subchondral osteoclast activity provides a mechanistic basis for the enhanced analgesic efficacy of LAC in osteoarthritis.

### 2.5 LAC Inhibits Sensitization and Proliferation of the Dorsal Root Ganglia

Synovitis is considered the primary cause of OA joint pain [46, 47]. On the one hand, polarization of synovial macrophages toward the pro‐inflammatory M1 phenotype reprograms cellular metabolism from oxidative phosphorylation to glycolysis, leading to lactic acid accumulation and acidification of the joint microenvironment. This acidic milieu activates acid‐sensing ion channel subunit 3 (ASIC3) and transient receptor potential vanilloid 1 (TRPV1), which are highly expressed on synovial sensory neurons and nerve endings, thereby promoting neuronal excitation and pain transmission [48, 49]. On the other hand, inflammatory mediators such as NGF released by M1 macrophages can directly stimulate aberrant sensory nerve sprouting within the joint, further amplifying pain sensitivity [50, 51]. Based on these mechanisms, we next investigated whether LAC could attenuate neuronal excitability and suppress pain‐related neural responses.

We first assessed intracellular pH changes in RAW264.7 cells from different treatment groups using the pH‐sensitive probe pHrodo Red AM. As shown in Figure 5A, LPS stimulation significantly reduced intracellular pH in RAW264.7 cells compared with the Control group. In contrast, treatment with LDH and LAC reduced fluorescence intensity by 15.7% and 24.5%, respectively, relative to the LPS group, indicating effective neutralization of intracellular acidity (Figure 5B). These results suggest that both LDH and LAC can buffer excess H^+^ generated by enhanced glycolysis in M1 macrophages, likely due to the abundant hydroxyl groups present in LDH‐based formulations. Notably, LAC exerted a stronger alkalizing effect than LDH alone, consistent with its ability to promote M1‐to‐M2 macrophage repolarization and further mitigate glycolytic acid production.

**FIGURE 5:**
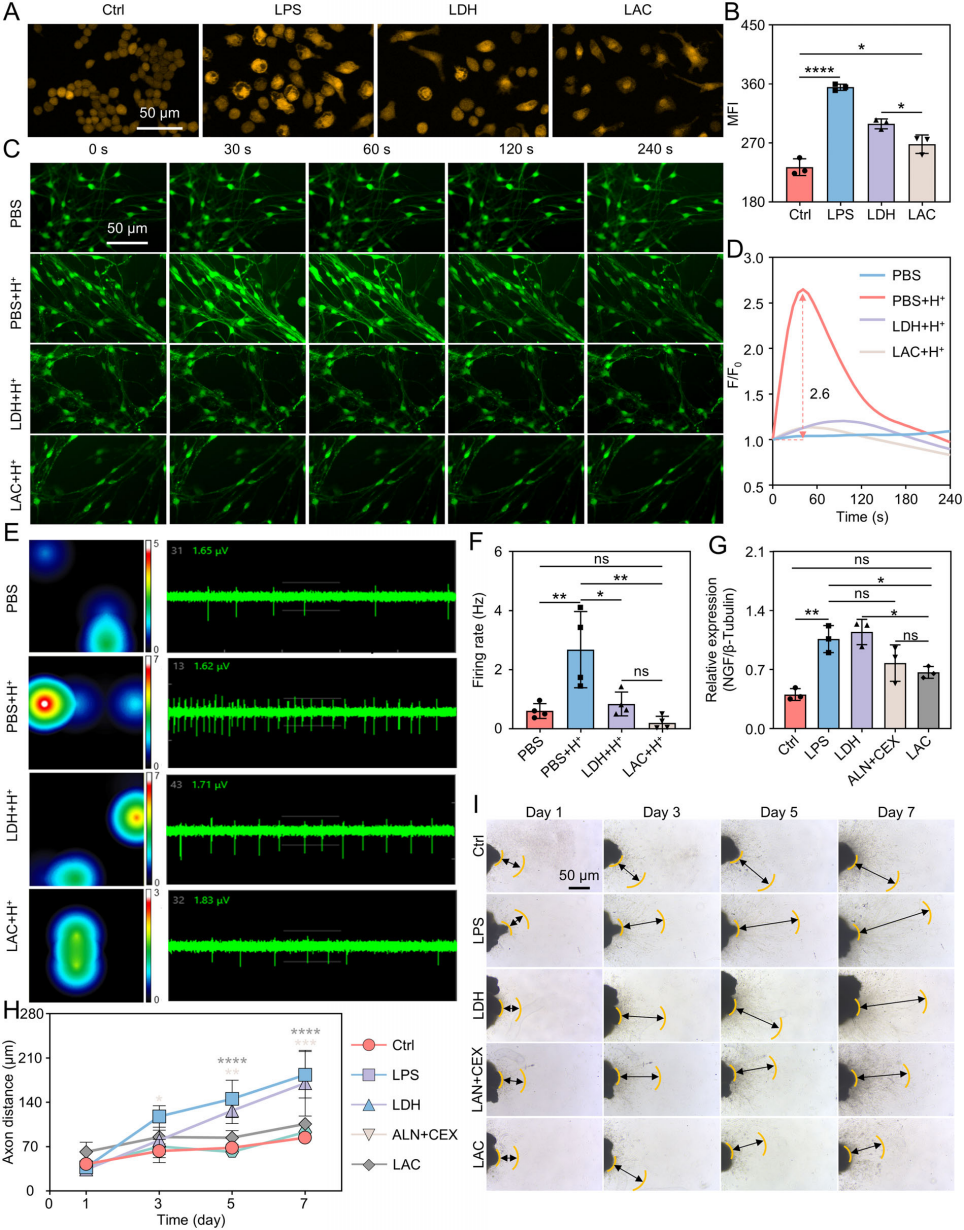
H^+^ signals and axon regeneration are blocked by LAC. (A) Measurement of intracellular pH with pHrodo Red AM and (B) the corresponding quantification of the mean fluorescence intensity within different treatment groups. (C) Calcium imaging of DRG cells treated with PBS, H^+^, LDH + H^+^, and LAC + H^+^ at 0, 30, 60, 120, and 240 s. (D) Calcium intensity change curve of DRG cells after treated with PBS, H^+^, LDH + H^+^, and LAC + H^+^. (E) Representative MEA recordings of spontaneous firing activity under control conditions and following treatment with LPS, LDH, or LAC. The firing rate at each active electrode is color coded, with red, orange, and yellow indicating higher firing rates and green and blue indicating lower firing rates. (F) Corresponding quantification of MEA data showing the mean firing rate for each condition. (G) Relative expression of NGF in the indicated groups. (H) Quantification of the relative axon distance of DRG explants in the indicated group. (I) The growth of axons in DRG cells co‐cultured with M1 macrophages in the indicated conditions. *n* = 3, mean ± SD. The significant differences are determined by one‐way ANOVA analysis with Tukey's multiple comparison test. *p < 0.05, **p < 0.01, ***p < 0.001, ****p < 0.0001, ns stands for no significant difference.

To determine whether attenuation of macrophage‐associated acidification translates into reduced neuronal excitability, we next monitored intracellular calcium dynamics in DRG neurons. DRG neurons isolated from 3‐week‐old Sprague–Dawley rats were cultured in vitro for 7 days before experimentation. Upon addition of H^+^ to the culture medium, DRG neurons exhibited a rapid increase in intracellular Ca^2^^+^ levels, reaching approximately 2.6‐fold above baseline within 30 s, as detected using the Fluo‐4 AM probe, followed by a gradual return to baseline (Figure 5C). In contrast, DRG neurons pretreated with LDH or LAC showed no significant changes in Ca^2^^+^ fluorescence intensity within 240 s after H^+^ challenge, indicating effective suppression of acid‐induced calcium influx (Figure 5D). Furthermore, neuronal excitability was evaluated using multielectrode array (MEA) recordings to assess spontaneous firing activity in rat DRG neurons. Baseline firing rates were recorded before treatment, with untreated neurons serving as controls. Application of H^+^ alone induced a marked increase in neuronal firing frequency (4.43‐fold relative to baseline). In contrast, co‐application of LDH or LAC significantly attenuated the H^+^‐induced elevation in spontaneous firing rates (Figure 5E,F). Collectively, these findings demonstrate that LDH‐based formulations, particularly LAC, could effectively reduce acid‐induced neuronal excitability by neutralizing excess H^+^ and mitigating macrophage‐driven acidification, thereby providing a mechanistic explanation for their analgesic effects in OA.

Subsequently, the effect of LAC on axonal growth mediated by macrophage polarization was further investigated using a coculture system of RAW264.7 macrophages and DRG neurons (Figure S33). As shown in Figure 5G and Figure S34, the expression of NGF in LPS‐induced M1 macrophages was increased by approximately 1.63‐fold compared with the Control group. In contrast, LAC treatment markedly reduced NGF expression, restoring it to levels comparable to those observed in the Control group. Consistent with these changes, DRG explants cocultured with LPS‐induced macrophages exhibited robust axonal outgrowth, with axon length increasing by approximately 3.92‐fold over a 7‐day culture period, reflecting the pro‐neuritogenic effect of M1 macrophage‐derived NGF. Notably, ALN + CEX and LAC treatment significantly attenuated DRG axonal elongation. By Day 7, the average axon length in the ALN + CEX‐ and LAC‐treated group was reduced to 57.8% and 50.7%, respectively, of that observed in the LPS group (Figure 5H,I). These results indicate that suppression of macrophage‐derived NGF is sufficient to restrain inflammation‐associated axonal growth, and suggest that the CEX component (through its immunomodulatory effects on macrophages) plays a major role in this process, while incorporation into the LDH carrier (LAC) further enhances the overall inhibitory efficacy. Together with its ability to neutralize macrophage‐derived H^+^ and dampen neuronal excitability, these effects provide a complementary mechanistic explanation for the analgesic efficacy of LAC in osteoarthritis.

### 2.6 Pain‐Relieving Mechanism of LAC In Vivo

Guided by in vitro evidence and transcriptome sequencing, we further explored the role of LAC in alleviating pain in MIA mice by reducing soluble NGF, netrin‐1, and H^+^ levels in the OA environment. Initially, NGF expression in the joint cavity of mice in different groups was detected through immunohistochemistry. Compared with the Sham group, the expression of NGF in the MIA group increased significantly, whereas its expression in the CEX, LC, and LAC treatment groups decreased to varying degrees. Among them, the effect in the LAC group was far superior to that of the other groups, reaching a level similar to that of the Sham group (Figure 6A,B). Subsequently, through immunofluorescence staining, we could clearly observe high expression of netrin‐1 in the bone marrow cavity of mice in the MIA group, whereas in the LAC group, this decreased by 43.2% compared with that in the MIA group, reaching the same level as that in the Sham group. This indicated that LAC inhibited the activity of osteoclasts to reduce netrin‐1 secretion in the bone marrow cavity (Figure 6C). Moreover, the effect of LAC was much greater than that of the CEX, LDH, and LC groups in terms of reducing netrin‐1 secretion. Finally, the pH values of joint fluids of the different groups were analyzed at the end of treatment. As shown in Figure 6D, the synovial fluid pH of the MIA group dropped from 7.28 to 6.95 when compared to that of the Sham group, whereas the synovial fluid pH of the LAC group (∼7.14) neared that of the Sham group, indicating that LAC can eliminate excess H^+^ to prevent pain sensitization. The pH values of the other groups were lower than those of the LAC group to varying degrees because LAC can eliminate H^+^ through dual effects: directly neutralizing H^+^ and inducing macrophage immune rearrangement.

**FIGURE 6:**
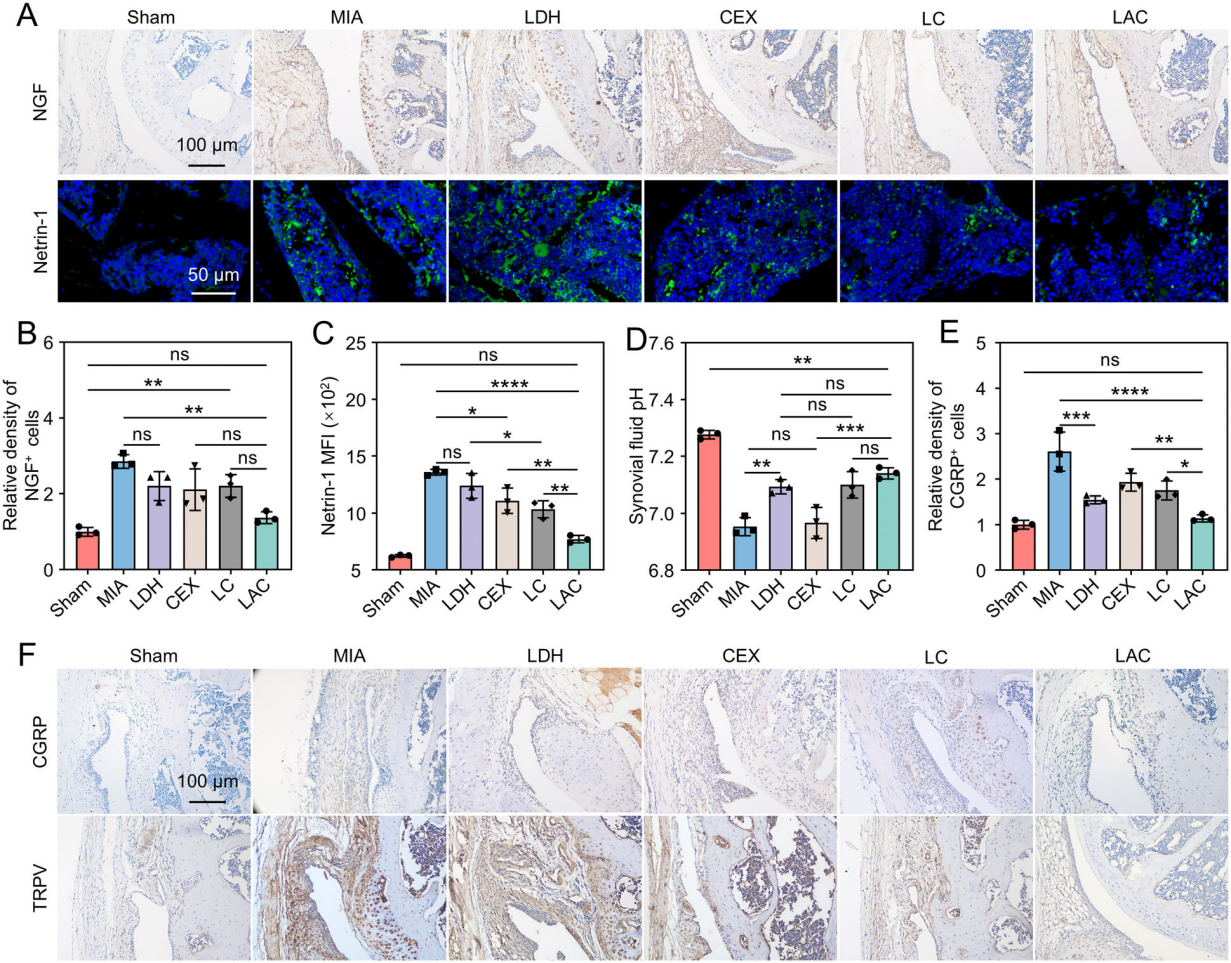
Pain‐relieving effect of LAC in the MIA mouse model. (A) Immunohistochemical staining of NGF and immunofluorescence staining of netrin‐1 in mice with different treatments. (B) Quantification of the relative density of NGF^+^ cells in mice with different treatments. (C) Quantification of the fluorescence intensity of netrin‐1 in mice with different treatments. (D) Synovial fluid pH in mice with different treatments. (E) Quantification of the relative density of CGRP^+^ cells in mice with different treatments. (F) IHC staining of CGRP and TRPV in the knee joints of mice with different treatments. *n* = 3, mean ± SD. The significant differences are determined by one‐way ANOVA analysis with Tukey's multiple comparison test. *p < 0.05, **p < 0.01, ***p < 0.001, ****p < 0.0001, ns stands for no significant difference.

In view of the fact that LAC can significantly downregulate three key soluble neurosensitizing factors in the joint cavity of OA mice, we then tested the two markers, TRPV1 (response to capsaicin and thermal stimulation; a cation channel that induces pain in acidic environments) and CGRP (calcitonin gene‐related peptide; a neuropeptide that promotes synaptic transmission of pain signals and nerve excitation), in the joint cavity of mice in different groups. Compared with the Sham group, the expression distribution of TRPV1 and CGRP in the synovium of mice in the MIA group was increased by 4.13 and 1.61 times, respectively, indicating that MIA mice developed severe joint pain (Figure 6E,F and Figure S35). The expression of TRPV and CGRP after LAC treatment decreased by 53.0% and 56.3%, respectively, compared to that in the MIA group, indicating that LAC can significantly reduce the distribution of pain sensory neurons into the joint cavity and relieve pain. The expression of TRPV and CGRP in the other groups was lower than that in the MIA group; however, the effect was far worse than that in the LAC group. Immunofluorescence images of CGRP in the bone marrow cavity of mice further indicated that the expression of CGRP in the LAC group decreased by 63.1% compared to that in the MIA group (Figure S36). These results confirm that LAC can effectively eliminate the three joint soluble nerve sensitization factors, and its effect is superior to that of other drugs that only act on a single factor.

### 2.7 Therapeutic Effect of LAC in the Monoiodoacetate‐Induced Osteoarthritis Model

Next, we verified the therapeutic effect and mechanism of action of LAC in MIA mice. OA mainly involves synovial inflammation, cartilage degradation, and subchondral bone destruction [52, 53, 54]. Initially, the synovium of the joints of each group of mice was collected for hematoxylin and eosin (H&E) staining after treatment. As shown in Figure 7A, compared with the Sham group, the thickness of the synovium in the MIA group was significantly increased, and nuclei of its synovial cells were significantly larger and more elongated, indicating that the synovium experienced hyperplasia and was severely inflamed in the MIA group. In comparison, synovial membrane thickness and the size of synovial cells in the LAC group were significantly lower than those in the MIA group. Further, by determining the max synovitis score, that in the LAC group dropped by nearly 37.0% compared with that in the MIA group. Although synovitis scores of the CEX, LDH, and LC groups also decreased to varying degrees compared with those of the MIA group, the differences were not significant, illustrating the favorable effect of LAC in relieving synovitis and the significant improvement when compared with CEX, LDH, and LC (Figure 7B). Next, the extent of cartilage damage in the joints of each group of mice was analyzed using safranin O/fast green (SO&FG) staining. Compared to the Sham group, the MIA, LDH, CEX, and LC groups showed varying degrees of cartilage erosion and loss of collagen and proteoglycans on the cartilage surface. In contrast, LAC showed a significant cartilage‐protective effect (Figure 7C). Osteoarthritis Research Society International (OARSI) scores of the LAC group dropped by nearly 66.7% compared to those of the MIA group, which were at the same level as those of the Sham group. Although OARSI scores of the CEX and LC groups also showed a certain degree of decline compared with those of the MIA group, the difference was not significant (Figure 7D). For a more accurate assessment of the changes in the trabecular and cortical bone architecture, a quantitative analysis of the bone parameters, including bone surface/bone volume (BS/TV), trabecular pattern factor (Tb.Pf), trabecular thickness (Tb.Th), trabecular number (Tb.N), trabecular bone separation (Tb.Sp), and bone surface/bone volume (BS/BV) based on the data from the micro‐computed tomography (micro‐CT) (Figure S37). The quantitative results suggested that, compared to the Sham group, the bone volume per tissue volume (BV/TV) values for the MIA group decreased from 39.3% to 32.3%. In contrast, LAC‐treated mice exhibited BV/TV values of 38.3%, similar to those of the Sham group. Moreover, LAC treatment led to a notable recovery in BV/TV, Tb.Pf, Tb.Th, and BS/BV, demonstrating its efficacy in alleviating OA.

**FIGURE 7:**
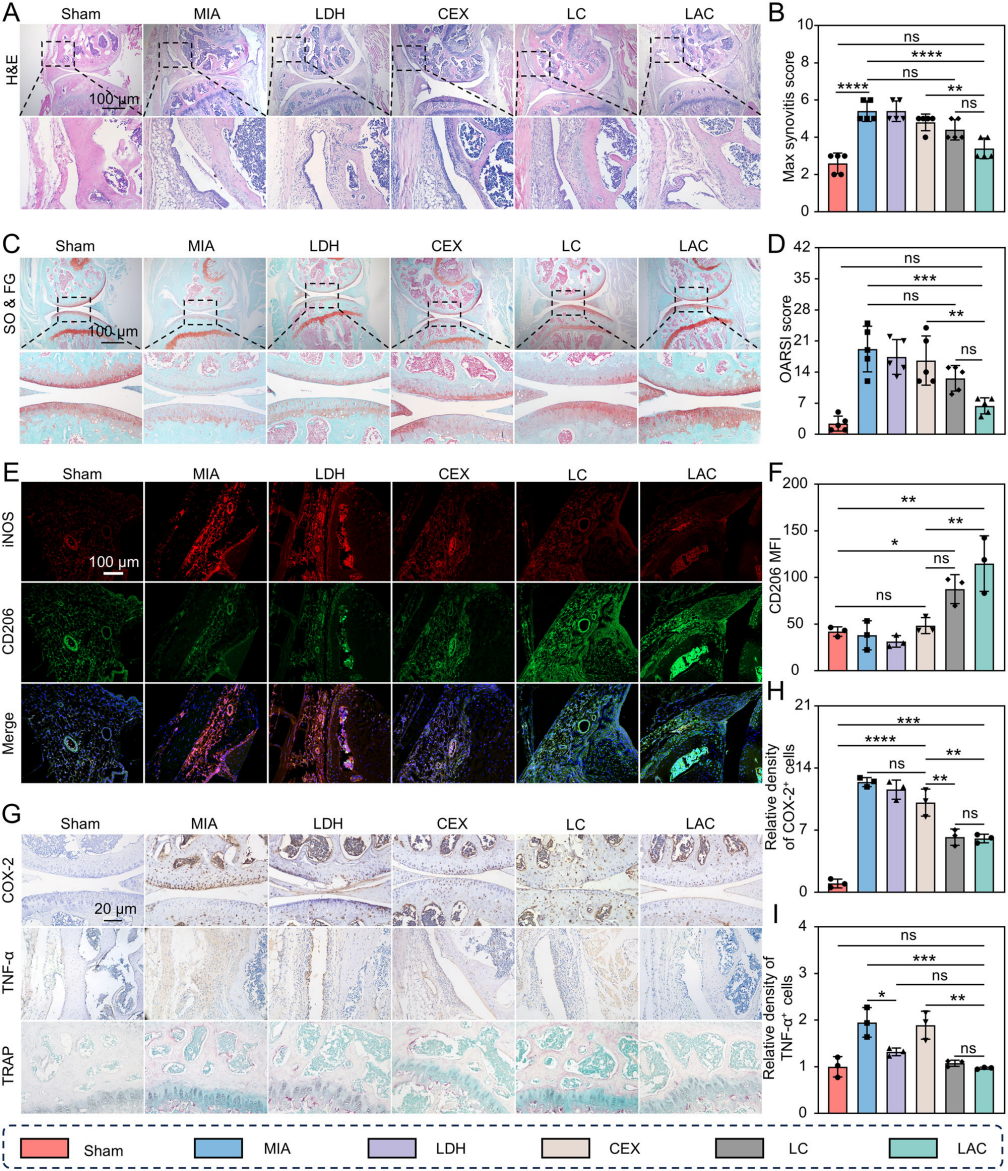
Therapeutic effect of LAC in the monoiodoacetate mouse model. (A) H&E staining of the joint from MIA mice after different treatments. (B) Quantitative analysis of the synovitis score. (C) Representative images of SO&FG‐stained knee joint sections from MIA mice with different treatments. (D) The severity of OA‐like lesions was analyzed using the OARSI scoring system. (E) Immunofluorescence imaging of iNOS (red)/CD206 (green) within the knee joints of mice with different treatments. (F) Corresponding quantification of the MFI of CD206 in the synovium of mice with different treatments. (G) Immunohistochemical staining of COX‐2, TNF‐α, and TRAP in the knee joints of mice with different treatments. (H,I) Quantification of the relative density of COX‐2^+^ (H) and TNF‐α^+^ (I) cells in the knee joints of mice with different treatments. *n* = 5 in B and D, and *n* = 3 in F, H, and I, mean ± SD. The significant differences are determined by one‐way ANOVA analysis with Tukey's multiple comparison test. **p < 0.01, ***p < 0.001, ****p < 0.0001, ns stands for no significant difference.

The therapeutic mechanism of LAC was further explored in MIA mice. The phenotype of macrophages in the synovium of each group of mice was detected via immunofluorescence using iNOS and CD206 costaining. Compared with the MIA and LDH groups, iNOS levels in the synovium of mice were significantly reduced in the CEX, LC, and LAC groups. CD206 levels in the LAC group were 3.02 and 1.31 times those in the MIA and LC groups, respectively (Figure 7E,F and Figure S38), suggesting that LAC promotes the phenotypic transformation of M1 macrophages to the M2 type in the synovium. LAC‐induced phenotypic transformation of macrophages is closely related to inhibition of COX‐2 expression. As shown in Figure 7G, the expression of COX‐2 in mice in the MIA group was significantly upregulated, whereas that in the CEX group was slightly downregulated, but not significantly. In comparison, the expression of COX‐2 in the LC and LAC treatment groups decreased by 50.0% and 51.2%, respectively, compared to that in the MIA group (Figure 7H). Similar results were obtained for the detection of TNF‐α, in which its expression levels in the synovium of mice treated with LC and LAC were decreased by 44.8% and 50.4%, respectively, compared to those in the MIA group (Figure 7I). Immunohistochemical TRAP staining further suggested that the number of TRAP^+^ osteoclasts in the subchondral bones of mice in the LAC group decreased by 2.26‐fold compared to that in the MIA group, reaching a level similar to that in the Sham group, indicating that LAC can significantly inhibit osteoclast activity (Figure S39). These results confirm that LAC significantly alleviates synovial inflammation, cartilage loss, and subchondral bone destruction by promoting the phenotypic transformation of synovial macrophages and inhibiting osteoclast activity.

### 2.8 Treatment Effect of LAC in the Destabilized Medial Meniscus‐Induced Osteoarthritis Model

Another OA model, the sports injury‐induced OA model, was constructed to explore the universality of LAC treatment. To this end, 12‐week‐old male C57BL/6J mice underwent destabilization of the medial meniscus (DMM) surgery to establish an OA mouse model [55, 56]. As shown in Figure 8A, each group of mice was treated once a week with an intra‐articular injection for a total of 8 weeks. First, the effect of LAC on the pathological progression of the DMM mouse model was investigated. Through evaluating H&E staining of the synovium of the OA joints of each group of mice, the DMM group showed obvious synovial hyperplasia compared to that in the Sham group, where LAC treatment significantly alleviated the symptoms (Figure 8B). By staining the cartilage with SO&FG, we observed that mice in the DMM group had severe cartilage wear compared to those in the Sham group, where LAC treatment significantly alleviated this (Figure 8C). Specifically, synovitis scores and OARSI score in the LAC group decreased by 24.2% AND 65.8%, respectively, compared to those in the DMM group (Figure 8D,E). Moreover, the expression level of matrix metallopeptidase 13 (MMP13) in the LAC group decreased by 1.37 times compared to that in the DMM group, as measured via immunohistochemistry, reaching the same level as that in the Sham group (Figure 8F,G). Accordingly, the expression level of aggrecan (AGG) was upregulated to 2.43 times that in the DMM group (Figure 8H). These results demonstrate the protective effect of LAC on OA cartilage. We comprehensively evaluated the osteophytes and meniscal calcification in mice from each treatment group using micro‐CT. Compared to the Sham group, mice in the DMM group formed larger periarticular osteophytes, the volume and surface area of ​the osteophytes increased significantly, and the degree of meniscus calcification also increased significantly. Fortunately, the osteophyte number, volume, and meniscus calcification degree of mice in the LAC group decreased by 0.33, 3.01, and 0.43 times, respectively, compared with those in the DMM group (Figure 8I,J and Figure S40). Finally, the effect of LAC on the alleviation of joint pain in the DMM‐induced OA model was explored. Compared with the continuously declining bipedal balance and paw withdrawal thresholds of mice in the DMM group, the bipedal balance (Figure 8K) and paw withdrawal thresholds (Figure 8L) of mice in the LAC group recovered to 88.5% and 71.9% of the normal levels, respectively, at the end of the treatment period. In summary, these results indicate that LAC alleviates OA progression and inhibits pain in the DMM‐induced OA model. Therefore, LAC has favorable universality for the treatment of OA.

**FIGURE 8:**
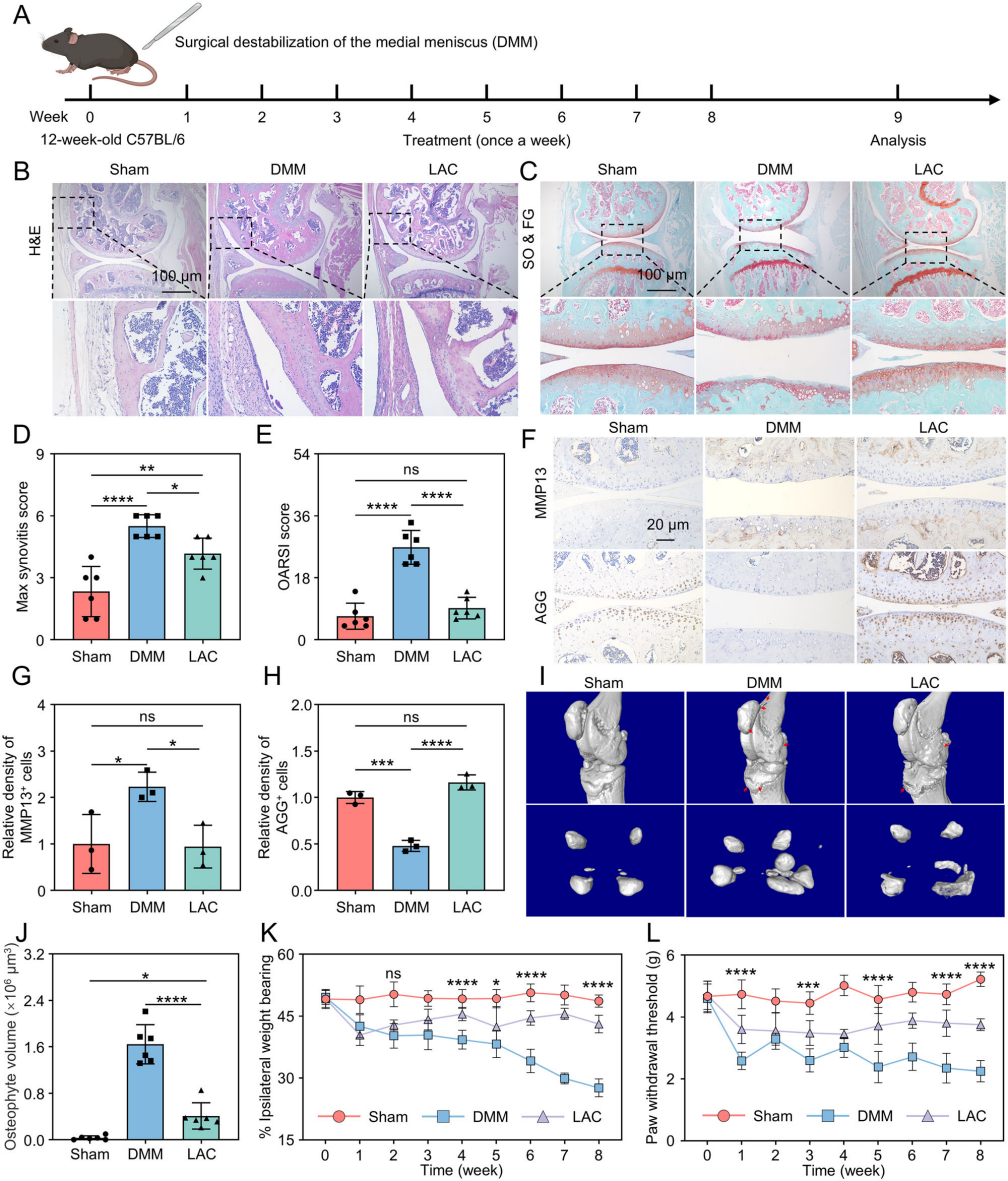
Therapeutic effect of LAC in a destabilized medial meniscus mouse model. (A) The establishment and therapeutic regimen of the DMM mouse model. (B) H&E staining of the joint from DMM mice after different treatments. (C) Representative images of safranin O & fast green (SO&FG)‐stained knee joint sections from DMM mice with different treatments. (D) Quantitative analysis of the synovitis score. (E) The severity of OA‐like lesions was analyzed using the OARSI scoring system. (F) Immunohistochemical staining of MMP13 and AGG in knee joints. (G,H) The quantification of MMP13‐ (G) and AGG‐ (H) positive cells compared with total cells. (I) Representative micro‐CT 3D rendering images of the knee joint (upper) and calcified meniscus and synovial tissues (lower) in the indicated groups. The osteophyte sites were highlighted with red arrows. (J) Osteophyte volume in the knee joints of mice with the indicated treatments. (K) Monitoring the weight‐bearing asymmetry for 6 weeks. (L) Paw withdrawal thresholds of the ipsilateral hind paws. *n* = 6 in D,E and J–L, *n* = 3 in G and H, mean ± SD. The significant differences are determined by one‐way ANOVA analysis with Tukey's multiple comparison test. *p < 0.05, **p < 0.01, ***p < 0.001, ****p < 0.0001, ns stands for no significant difference.

### 2.9 Biocompatibility of LAC

Given that LAC is composed of Mg/Al‐LDH loaded with CEX and ALN, its intra‐articular degradation inevitably leads to the local and systemic exposure of multiple components, including Mg^2^^+^ and Al^3^^+^ ions as well as the released drugs. In particular, the long‐term biosafety of Al‐containing nanomaterials has raised regulatory and toxicological concerns. Therefore, a comprehensive in vivo biosafety evaluation was conducted to assess the overall biological impact of LAC and its degradation products under repeated administration conditions.

Using healthy mice as controls, LAC was injected into the joint cavity of DMM‐induced OA mice once a week for 8 weeks. Thereafter, H&E staining of major organs (heart, liver, spleen, lung, and kidney), liver function indicators, such as alanine aminotransferase (ALT) and aspartate aminotransferase (AST), and renal function indicators, such as blood urea nitrogen (BUN) and creatinine, were tested in each group of mice (Figure S41A). As shown in Figure S41B, LAC treatment did not cause histopathological abnormalities or lesions in these organs. Moreover, no significant differences in the above serum biochemical parameters were observed among mice in the healthy, DMM, and LAC groups (Figures S41C–E and S42). Finally, complete blood cell count tests of white blood cells, red blood cells, platelets, etc., further confirmed no significant changes among different treatment groups (Figures S41F–I and S43). Collectively, these results indicate that repeated intra‐articular administration of LAC does not induce detectable systemic toxicity, organ damage, or hematological abnormalities, suggesting favorable biocompatibility and biosafety of the LAC system as a whole within the investigated treatment duration.

## 3 Conclusion

In this study, we designed and fabricated a multifunctional LDH‐based nanosheet formulation (LAC) by co‐loading the anti‐inflammatory drug, CEX, and the anti‐osteoporosis agent, ALN, onto LDHs, aiming to concurrently remodel the inflammatory synovium and aberrant subchondral bone and thereby disrupt pathological OA nerve–tissue crosstalk. Upon intra‐articular administration, the acidic synovial microenvironment triggered the responsive release of CEX, which effectively repolarized pro‐inflammatory M1‐like macrophages toward an immunoregulatory M2‐like phenotype and suppressed the production of key algogenic mediators, including TNF‐α and NGF. With the gradual degradation of LAC, ALN was subsequently released and penetrated the subchondral bone, inhibiting osteoclast formation and bone resorption while reducing netrin‐1 expression. The coordinated downregulation of NGF and netrin‐1 effectively limited axonal guidance and aberrant nerve innervation into cartilage, while the abundant hydroxyl groups of LDH conferred a buffering capacity to neutralize excessive H^+^, collectively attenuating nociceptor excitation and OA pain behaviors.

Compared with conventional OA therapies that often suffer from delayed onset, limited durability, or systemic side effects, LAC offers a locally confined, multimodal strategy with rapid pharmacological activation and sustained joint retention. Notably, the LDH framework is composed of physiologically relevant metal elements, and the doses of CEX delivered intra‐articularly are substantially lower than those used in systemic administration, contributing to favorable biosafety profiles. Nevertheless, OA is a multifactorial and heterogeneous disease, frequently accompanied by aging‐related and metabolic comorbidities, which may influence therapeutic responsiveness. Moreover, the present work primarily addresses early‐stage OA pathology, whereas most clinical cases are diagnosed at more advanced stages. Future studies in late‐stage OA models, as well as evaluations of manufacturing consistency and long‐term translational feasibility, will be essential. Taken together, our findings establish LAC as a promising platform for integrated modulation of synovial inflammation, subchondral bone remodeling, and neural sensitization, offering a potential therapeutic avenue for effective OA pain management.

## 4 Materials and Methods

### 4.1 Materials and Cell Lines

DMEM with 4.5 g glucose, penicillin/streptomycin (P/S), 0.25% trypsin‐EDTA, and fetal bovine serum (FBS) were purchased from Gibco (Grand Island, NY). RAW264.7 cells were cultured in DMEM supplemented with 10% FBS (heat inactivated).

Aluminum nitrate nonahydrate [Al(NO_3_)_3_·9H_2_O] (E2117032) were obtained from Aladdin. Magnesium chloride hexahydrate [MgCl_2_·6H_2_O] (C11151130), ALN (C13688051), CEX (C14572997), sodium hydroxide (NaOH, C13221232), BODIPY (C13886665), and dimethyl sulfoxide (DMSO, C12069520) were purchased from Macklin. Sulfo‐Cyanine 7 NHS (D10018) was purchased from Duofluor Inc.

### 4.2 Preparation and Characterization of LAC

First, the magnesium‐aluminum double‐layered hydroxide (LDH) was synthesized. Then15 mmol MgCl_2_·6H_2_O and 5 mmol Al(NO_3_)_3_·9H_2_O were mixed and dissolved in 50 mL ultrapure water with carbon dioxide removed. Two hundred milliliter NaOH solution (150 mM) was slowly added to the prepared salt solution and stirred at room temperature for 10 min. Subsequently, the supernatant was discarded by 11,000 rpm centrifugation for 10 min, while the precipitate was washed three times and then redispersed in 120 mL of ultrapure water. The slurry was transferred into an autoclave and aged at 100°C for 16 h. The products LDH were washed with ultrapure water and ethanol three times, and dispersed into 30 mL ethanol at 4°C for storage. In addition, 1 mL of acquired LDH was centrifuged and dried to measure the accurate concentration.

Second, ALN and CEX were sequentially loaded into LDH prepared as above: 5 mg ALN was dissolved in ultrapure water. At the same time, 30 mg of the previously prepared LDH were collected by centrifugation, and the supernatant was discarded. Three milliliter of ALN and 8 mL of LDH were added to a 25 mL round‐bottomed flask, rinsed with 1 mL ultrapure water. The mixture was stirred at low temperature for 10 h and centrifuged (13,000 rpm for 10 min) to obtain ALN‐loaded LDH (LA). LA was dispersed into 11 mL ultrapure water, 1 mL of which was centrifuged and dried to measure the accurate concentration. The remaining 10 mL LA was redispersed in 2 mL DMSO after centrifuge and an equal amount of CEX dissolved in DMSO was added, forming the accurate 3 mL reaction system. The mixture was stirred for 10 h and centrifuged. After collecting the supernatant, LAC was obtained after washing with ultrapure water.

Finally, the synthesized LDH and LAC were characterized. TEM images were captured using a TECNAI G2 high‐resolution transmission electron microscope. Zeta‐potential testing was carried out using a Zetasizer Nano ZS90. XPS measurements employed a VG ESCALAB MKII spectrometer. Deconvolution of the narrow‐scan XPS spectra was done with Thermo Avantage software (version 5.991). XRD was measured by the Rigaku Ultima IV x‐ray diffractometer instrument.

### 4.3 H^+^ Depleting Efficiency and LAC Degradation in Solution

Eight milligram LAC was collected by centrifugation and redispersed in 10 mL of pH 7.4 buffer solution (PBS) and pH 6.6 PBS, respectively. The LAC in PBS was stirred at room temperature to fully contact with the H^+^ for 7 days, with the pH of the PBS solution measured by an electronic pH meter at 0, 0.5, 1, 2, 4, 8, 12, 24, 48, 72, 96, 120, 144, and 168 h. The solution samples of the mixture were collected at 0, 24, and 168 h to observe LAC degradation in TEM.

### 4.4 CEX Loading and Releasing Efficiency in Solution

To measure the drug loading and release efficacy of LAC, a standard concentration curve of CEX was first prepared using UV spectrophotometry. Supernatant from the drug loading process was collected, and the UV absorption spectrum of the supernatant was measured. The concentration of CEX in the supernatant was calculated using the standard curve. The drug loading efficiency was calculated using the formula “(total mass of CEX − supernatant CEX concentration × reaction volume)/total mass of LDH × 100%.”

As for LAC's pH release characteristics, 2 mg LAC was collected by centrifugation and redispersed in 30 mL of pH 7.4 and pH 6.6 PBS (containing 0.1% SDS), respectively, shaken in the same thermostat water bath (100 rpm, 37°C). Take 5.0 mL of release medium from centrifuge tubes at set time points (0.5, 1, 2, 4, 8, 24, 48, 72, 96, 120, 144, and 168 h.), and add buffer solution of the same volume, temperature, and pH. After centrifuging the sample (11,000 rpm, 1 min) to obtain the supernatant, the concentration was determined using UV and referencing the standard curve of CEX loading. Then calculate the release rate at each time point based on the release rate formula “[supernatant CEX concentration × 30 mL × (6/5)*^n^* ^− 1^]/[total mass of LAC × drug loading)] × 100%, “*n*” represents the *n*th sampling.” Finally, draw the cumulative release curve based on the release rate at each time point.

### 4.5 ALN Loading and Releasing Efficiency in Solution

Due to the absence of an absorbing chromophore, ALN cannot be directly detected using UV spectrophotometry. The derivatization of ALN with OPA plus 2‐Mercaptoethanol (2ME) endows it with the absorption maximum at 330 nm. According to the known reaction between OPA and amino compounds, the primary amine group of ALN is believed to react with the two aldehyde groups of OPA to yield an isoindole derivative. The specific operation is as follows: first, prepare the derivatization reagent OPA/2ME solution. Weigh 50 mg of OPA and dissolve it in 5 mL of 0.05 M NaOH, then add 250 µL of 2ME solution. Finally, add 44.75 mL of 0.05 M NaOH to the mixture. For the sample to be tested, take 0.4 mL of the sample and add 0.4 mL of 0.1 M NaOH. Then, add 0.32 mL of the freshly prepared derivatizing reagent (OPA/2ME) solution. Finally, add 2.88 mL of NaOH. After 60 min, detect the sample using UV spectroscopy. Subsequently, a standard concentration curve of ALN was prepared using UV spectrophotometry and characteristic absorption of derivatives at 330 nm. Supernatant from the drug loading process was collected, and the UV absorption spectrum of the supernatant was measured. The concentration of ALN in the supernatant was calculated using the standard curve. The drug loading efficiency was calculated using the formula “(total mass of ALN − supernatant ALN concentration × reaction volume)/total mass of LA × 100%.”

As for LA's pH release characteristics, 15 mg LA was collected by centrifugation and redispersed in 10 mL of pH 7.4 and pH 6.6 PBS, respectively, shaken in the same thermostat water bath (100 rpm, 37°C). Take 2.0 mL of release medium from centrifuge tubes at set time points (24, 48, 72, 96, 120, 144, and 168 h.), and add buffer solution of the same volume, temperature, and pH. After centrifuging the sample (13,000 rpm, 10 min) to obtain the supernatant, the concentration was determined using the OPA/2ME and UV method and referencing the standard curve of ALN loading. Then calculate the release rate at each time point based on the release rate formula “[supernatant ALN concentration × 10 mL × (5/4)*^n^* ^− 1^]/total mass of LA × drug loading × 100%, “*n*” represents the nth sampling.” Finally, draw the cumulative release curve based on the release rates at each time point.

### 4.6 Western Blotting

All the samples were washed and lysed with RIPA buffer (Beyotime) supplemented with PMSF (Beyotime) and Phosphatase inhibitor cocktail (Beyotime) on ice to extract protein. The bicinchoninic acid protein assay kit (Beyotime) was used to measure the concentration of protein. Proteins in equivalent amounts were then separated on a 10% sodium dodecyl sulfate‐polyacrylamide gel electrophoresis (SDS‐PAGE) and transferred to the PVDF membrane, followed by blocking in TBS‐T solution containing 5% skim milk for 1 h. Subsequently, the membrane was incubated with primary antibody against COX‐2 (66351‐1‐Ig, Proteintech), iNOS (80517‐1‐RR, Proteintech), and CD206 (60143‐1‐Ig, Proteintech) overnight at 4°C. Then, secondary antibodies conjugated to horseradish peroxidase (HRP) were added for 1 h at room temperature, and the protein blots were visualized via x‐ray film. GAPDH (60004‐1‐Ig, Proteintech) and β‐actin (81115‐1‐RR, Proteintech) were employed as the protein loading control.

### 4.7 Flow Cytometric Analysis

RAW264.7 cells were pretreated with PBS, LDH (75 µg mL^−^^1^), or LAC (75 µg mL^−^^1^) for 24 h, followed by stimulation with LPS (1 µg mL^−^^1^) overnight. Thereafter, the cells were washed with PBS three times and blocked with 1% BSA for 10 min. After washing with PBS three times, the cells were incubated with anti‐F4/80‐FITC, anti‐iNOS‐PE, and anti‐CD206‐Alexa Fluor 647 for 1 h at room temperature. All the antibodies used above were purchased from BioLegend. Flow cytometric data acquisition was performed with NovoCyte software, and the data were processed using FlowJo software.

### 4.8 Macrophage Polarization Detection

Cells in different treatment groups were washed with PBS three times and fixed with 4% paraformaldehyde. After incubation with 0.1% Triton X‐100 for 10 min, the cells were blocked with 1% BSA, and then stained with anti‐iNOS (ab49999, ABCAM) and anti‐CD206 (60143‐1‐Ig, Proteintech) at 4°C overnight. After washing with PBS three times, the cells were incubated with Alexa Fluor 488‐conjugated antibody (ab150077, ABCAM) and CoraLite594‐conjugated antibody (SA00013‐3, Proteintech) at room temperature for 1 h. Nuclei were counterstained with DAPI (ab104139, ABCAM), and then the stained sections were imaged with a confocal microscope.

### 4.9 In Vitro Calcium Imaging of DRG Cells

Primary DRG cells were dissected from 3‐week‐old rats and cultured in Neurobasal‐A media supplemented with 50 ng mL^−^^1^ NGF, 1× GlutaMax, 1× B‐27 supplement, and 1% P/S according to the protocol provided by Professor Caroline. Briefly, rats were euthanized with CO_2_ and transcardially perfused with PBS to minimize blood contamination. The vertebral column is dissected, cleared of soft tissue, and split sagittally to expose the spinal canal. Thereafter, individual DRGs were carefully pulled out from the intervertebral foramina with fine tweezers and trimmed of attached spinal roots to reduce myelin debris. The collected ganglia undergo enzymatic digestion using a Collagenase A/Dispase II mix at 37°C for 30 min, followed by mechanical trituration through increasingly fine needles to generate a single‐cell suspension. The resulting neurons are then plated onto laminin‐coated surfaces and cultured in supplemented Neurobasal media mentioned above before experimental use. For calcium imaging, DRG cells were treated with PBS or LDH (200 µg mL^−^^1^) for 6 h, respectively. After washing with PBS three times, the cells were stained with Fluo‐4 (40704ES50, Yeasen) for 30 min. Thereafter, cells were washed with PBS three times. In the PBS + H+ and LDH + H+ groups, 10 µL 0.1 M HCl solution was dripped into the medium before observation. The fluorescence (λ_ex_ = 495 nm, λ_em_ = 516 nm) in different treatment groups was monitored under a fluorescence microscope (Zeiss).

### 4.10 Construction of MIA Mice and Related Treatments

The C57BL/6 mice were purchased from Hunan Silaikejingda Experimental Animal Co., Ltd., and all mice were housed in specific pathogen‐free (SPF) barrier conditions with a 12‐h light/dark cycle and provided with ample food and water. The research adhered to the ethical standards and welfare guidelines for laboratory animals. Approval for the experimental protocol was obtained from the Laboratory Animal Welfare and Ethical Committee at Central South University. All animal experiments have been approved by the Institutional Animal Care and Use Committee (IACUC), Xiangya Hospital, Central South University, and Xiangya School of Medicine, Central South University (approval no. XMSB‐2023‐0188). Approval for the experimental protocol was obtained from the IACUC, Hunan Prima Drug Research Center Co., Ltd. The ethical approval number was IACUC‐202202.

Animals were randomly assigned to different experimental groups. The monosodium iodoacetate (MIA) model is induced by a single intra‐articular injection of MIA (20 mg kg^−^^1^) into the right knee joint of 12‐week‐old mice, resulting in damage to the synovium and corresponding cartilage within 3 days. Furthermore, mice of the same age received an equivalent volume of physiological saline injected into the joint cavity as the normal control group. For the therapeutic efficacy study, LAC, LC, LDH, CEX (0.125 mg kg^−^^1^), or PBS with the same volume was intra‐articularly injected into the right knee of MIA mice once a week for 4 weeks. At the endpoint of the experiment, the mice were euthanized, and joints were harvested for further examination. In the toxicity verification experiment of LAC, peripheral blood, heart, liver, spleen, lung, kidney, and intestinal tissues were collected from the mice.

### 4.11 Immunohistochemical Analysis

To assess pathologic changes in articular cartilage and synovium, sensory nerve pain threshold, osteoclast differentiation, inflammation in mice and other related indicators, intestine joints samples were obtained from various groups of mice and immunohistochemistry, TRAP staining and other immunofluorescence (IF) staining were conducted, Sections were prepared from the tissues of three randomly chosen animals from each group.

The primary antibodies used in the IF staining included anti‐COX‐2, anti‐TRPV, anti‐ CGRP, anti‐ MMP13, anti‐AGG, anti‐TNF‐α, anti‐NGF, and anti‐netrin‐1.

SO&FG and H&E were used to evaluate the damage of cartilage and synovium separately. Using the OARSI semi‐quantitative histopathological scoring system, cartilage degeneration was graded on a scale of 0–6. Across all slides, the sum of the six highest scores was used to determine the severity of cartilage destruction. In grading synovitis, the total thickness of lining cells and cellularity were taken into account, where 0 indicates a lining cell layer 1–2 cells thick, 1 indicates 3–5 cells thick, 2 indicates 6–8 cells thick and/or mild increase in cellularity, and 3 indicates lining cells >9 cells thick and/or severe increase in cellularity.

After fixation with 4% paraformaldehyde and decalcification with EDTA, the tissues were embedded in paraffin and sectioned using a Leica microtome. Subsequently, deparaffinization and hydration were performed, followed by heat antigen retrieval and endogenous peroxidase blocking. Normal goat serum was added and incubated at room temperature for 30 min. The diluted primary antibodies were then added and incubated overnight at 4°C in a humid box. On the second day, fluorescent secondary antibodies were added and incubated at room temperature for 1 h in the humid box. After incubating with secondary antibodies, the slides were stained with DAB (ZLI‐9018, ZSGB‐BIO) and counterstained with hematoxylin. Finally, the images were observed and captured using a fluorescence microscope. Specific conditions were adjusted according to the manufacturer's instructions.

### 4.12 TEM of the Synovial Tissues

Synovial tissue from different groups was collected and fixed immediately in fresh TEM fixation for 1 day at 4°C. The samples were washed in PBS (pH 7.4) solution three times, 15 min each, and then fixed with 1% OsO_4_ in PBS for 2 h at room temperature, avoiding light. After removing OsO_4_ and washing three times in PBS, 15 min each, the samples were dehydrated at RT, followed by resin penetration and embedding, and polymerization. The resin blocks were cut to 60–80 nm thin on the ultramicrotome and floated out onto the 150 meshes cuprum grids with formvar film. Then the samplers were stained with 2% uranium acetate saturated alcohol solution for 8 min in the dark, rinsed in 70% ethanol three times, and then in ultrapure water three times. Finally, the samples were stained with 2.6% lead citrate, avoiding CO_2_ for 8 min, and rinsed with ultrapure water three times. The cuprum grids were put into the grid board and dried overnight at RT. The cuprum grids were observed under TEM (HITACHI HT7800/HT77000), and images were taken. Field emission TEM (Talos F200X) was used for TEM‐EDX scanning.

### 4.13 Micro‐CT Analysis

The knee joint specimens from both the LAC‐treated and control groups were securely mounted in ex vivo micro‐CT (IVIS, Spectrum, Bruker, Germany) to ensure proper positioning for imaging. The samples were rotated 360° at a speed of 0.4° s^−^^1^ to acquire multiple projections, resulting in raw data. Subsequently, the raw data were processed using SkyScan software to generate CT data. CTAn software was utilized to separate CT data for individual samples. Then, the DataViewer software was used to align the long axis of the defect site parallel to the Z‐axis and save images on the XY plane. Afterward, CTAn software was employed to select the region of interest (ROI) on the XY plane images for analysis, obtaining bone volume (BV) and tissue volume (TV). Finally, CTvol software was used for three‐dimensional reconstruction.

### 4.14 Pain Behavioral Assessment

Mechanical allodynia and hind paw weight distribution were assessed to evaluate pain‐related behavior in mice. The Electronic von Frey Anesthesiometer (IITC, Woodland Hills, CA) was used to measure changes in mechanical pain thresholds at different sites on mice. When a specific length of filament is vertically pressed onto the skin and pressure is applied continuously until the filament bends, the applied pressure can be calculated. The greater the pressure applied, the higher the pain threshold, primarily used to assess hyperalgesia and hypoalgesia. Next, the Double Hind Limb Balance Tester (Incapacitance Analgesia Meter, ITTC, Woodland Hills, CA) was used to assess pain in the hindlimbs of mice. The mice were positioned in a specific posture, and the machine began measuring the weight‐bearing of both hindlimbs. The instrument displayed the weight‐bearing values of both hindlimbs, with a greater difference indicating greater pain in the modeled hindlimb. Finally, weight‐bearing asymmetry was calculated as the percent difference in weight distribution between the hindlimbs. Baseline measurements were obtained immediately before intra‐articular injection or surgery.

### 4.15 In Vivo Imaging System

Noninvasive in vivo multispectral fluorescence imaging was performed to ensure the distribution of LAC after injection. MIA model were intra‐articular injected with Cy7‐labeled LAC (100 µL, 0.5 mg mL^−^^1^), and an equal amount of PBS was taken as a control. MIA models were anesthetized with isoflurane, and images were taken by the IVIS Spectrum system (Caliper, USA) at 1, 2, 3, 4, 5, and 7‐days post‐injection. Regions of interest were circled the organs, and the fluorescence intensity was analyzed by Living Image Software.

### 4.16 Osteoclastic Differentiation Evaluation In Vitro

RAW264.7 cells were cultured with 50 ng mL^−^^1^ RANKL, along with different treatments for 7 days. The culture medium was refreshed every 3 days. After incubation, the cells were fixed and stained using a TRAP staining kit. Cells displaying more than three nuclei and positive TRAP staining were identified and counted as mature osteoclasts. And Western blot was employed to detect netrin‐1 expression, which represents osteoclast activity.

### 4.17 Biocompatibility Assessment of LAC

A hematological analysis was conducted using whole blood samples to evaluate the biocompatibility of LAC. The parameters analyzed included blood routine, as well as liver and kidney function. To evaluate the long‐term toxicity, healthy mice were intra‐articularly injected with LAC for a duration of 30 days. Subsequently, changes in organ histology were analyzed using H&E staining.

### 4.18 DMM OA Model

DMM surgery was performed using 12‐week‐old male C57BL/6 mice at the right knee joint. Briefly, mice were anaesthetized with isoflurane supplied in a mouse anesthesia apparatus, followed by joint surgery on the right joint by sectioning the medial meniscotibial ligament. The sham operation with the control mice of the same age was performed with a similar incision at the right joint capsule without menisci ligament.

### 4.19 Statistical Analysis

All the experiments were carried out at least three times and expressed as mean ± standard deviation unless otherwise stated. An unpaired *t*‐test was leveraged to perform the statistical analysis in Graphpad Prism 9. The significant difference between experimental groups was analyzed using one‐way analysis of variance (ANOVA). Data were considered as significantly different at *p* < 0.05 (*), *p* < 0.01 (**), *p* < 0.001(***), *p* < 0.0001 (****), while ns indicates that there is no significant difference between the groups.

## Author Contributions

**Junyan Liu**: writing – original draft, review and editing, conceptualization, methodology, validation, formal analysis, data curation, visualization, investigation. **Feixiang Ding**: writing – review and editing, visualization, methodology. **Jiayi Li**: visualization, methodology. **Min Liu**: writing – review and editing, methodology. **Lingyu Su**: writing – original draft, software, data curation. **Zixi Wang**: validation, formal analysis, investigation. **Congying Pi**: validation, formal analysis, conceptualization. **Shilong Jiang**: validation, data curation. **Zhaokui Zeng**: validation, data curation. **Changjun Li**: writing – review and editing, conceptualization, supervision, methodology. **Chao Zeng**: writing – review and editing, conceptualization, supervision, visualization. **Guanghua Lei**: writing – review and editing, supervision, project administration, funding acquisition. **Kelong Ai**: writing – review and editing, conceptualization, supervision, project administration, funding acquisition.

## Conflicts of Interest

The authors declare no conflicts of interest.

## Ethics Statement

All animal experiments have been approved by the Institutional Animal Care and Use Committee (IACUC), Xiangya Hospital, Central South University and Xiangya School of Medicine, Central South University (approval no. XMSB‐2023‐0188). And approval for the experimental protocol was obtained from Institutional Animal Care and Use Committee (IACUC), Hunan Prima Drug Research Center Co., Ltd. The ethical approval number was IACUC‐202202.

## AI Statement

The authors used ChatGPT for language editing purposes only. No AI tools were employed in data analysis, figure preparation, or the generation of scientific content. All authors have reviewed and take full responsibility for the final manuscript.

## Supporting information




**Supporting File**: exp270233‐sup‐0001‐SuppMat.docx.

## Data Availability

The data that support the findings of this study are available from the corresponding author upon reasonable request.
