# Supplementary material for: Soluble Nanomedicine Against Soluble Neurosensitizers to Effectively Relieve Osteoarthritis Pain and Joint Damage
Source: Exploration (Beijing). 2026 Oct 8;6(5):70233. doi: 10.1002/exp2.70233 (PMC13650196; doi:10.1002/exp2.70233)
Supplement: Supplementary file 1 [file EXP2-6-70233-s001.docx]

Supplementary Materials

**Soluble nanomedicine against soluble neurosensitizers to effectively relieve OA pain and OA joint damage**

This file includes:

Supplementary Figures 1-43

**Figure S1.** (A) Hydrodynamic diameter distribution of LDH and LAC. (B) Zeta potential intensity of LDH and LAC. n = 3, mean ± SD.

**Figure S2.** XPS spectrum of LDH, LA and LAC.

**Figure S3.** C1s narrow XPS scan of LDH (A) and LAC (B).

**Figure S4.** Narrow XPS scan of LAC: F1s (A) and S2p (B).

**Figure S5.** O1s narrow XPS scan of LDH (A) and LAC (B).

**Figure S6.** Narrow XPS scan of LAC: N1s (A) and P2p (B).

**Figure S7.** Mg2p narrow XPS scan of LDH (A) and LAC (B).

**Figure S8.** Al2p narrow XPS scan of LDH (A) and LAC (B).

**Figure S9.** X-ray diffraction (XRD) pattern of LDH and LAC.

**Figure S10.** Concentration-dependent absorbance (A) and standard absorbance curve (B) of CEX.

**Figure S11.** Concentration-dependent absorbance (A) and standard absorbance curve (B) of ALN.

**Figure S12.** (A) Calibration curve for Mg^2+^ measured by AAS. (B) Mg^2+^ concentration within 10 mg mL^-1^ LAC. (C) Cumulative release curve of Mg^2+^ from LAC in pH 6.6 and pH 7.4 PBS within 7 days. n = 3, mean ± SD.

**Figure S13.** The fluorescence spectrum of LAC, Cy7, and Cy7-labeled LAC (Cy7-LAC).

**Figure S14.** Representative fluorescence images (A) and corresponding quantification (B) of the MFI of Cy-7 labeled LAC within knee joint at different time points post-injection. n = 3, mean ± SD. The significant differences are determined by one-way ANOVA with Tukey’s multiple comparison test. ***p* < 0.01, *****p* < 0.0001, ns stands for no significant difference.

**Figure S15.** (A) Transmission electron micrographs showing the uptake of LAC by macrophages in synovial tissue. Yellow arrows indicate the LAC particles. (B) Transmission electron microscopy-energy dispersive X-ray spectroscopy (TEM-EDX) scan of the synovial tissue from LAC-treated and untreated MIA models.

**Figure S16.** Immunofluorescence images and corresponding co-localization analysis of synovial tissues 5 days post-injection with Cy7-LAC.

**Figure S17.** Behavioral pain analysis. (A) Monitoring the weight-bearing asymmetry in 4 weeks. (B) Paw withdrawal thresholds of the ipsilateral hind paws in 4 weeks. n = 6, mean ± SD. The significant differences are determined by two-way ANOVA with Tukey’s multiple comparison test. **p* < 0.05, ***p* < 0.01, ****p* < 0.001, *****p* < 0.0001, ns stands for no significant difference.

**Figure S18.** Monitoring the weight-bearing asymmetry in 4 weeks. n = 6, mean ± SD. The significant differences are determined by two-way ANOVA with Tukey’s multiple comparison test. ****p* < 0.001, *****p* < 0.0001, ns stands for no significant difference.

**Figure S19.** Footprint test performed to assess the forelimb stride length (A) and sway length (B). n = 6, mean ± SD. The significant differences are determined by one-way ANOVA with Tukey’s multiple comparison test. **p* < 0.05, ***p* < 0.01, ****p* < 0.001, *****p* < 0.0001, ns stands for no significant difference.

**Figure S20.** Basic RNA-sequencing. (A) Venn diagram of whole-transcriptome RNA-seq analysis showing differentially expressed genes in pairs between Sham group, MIA group and LAC-treated group. (B)Volcano plot of differentially expressed genes determined between MIA group and LAC-treated group.

**Figure S21.** GO enrichment pathways for LAC compared to MIA group.

**Figure S22.** Relative expression of genes in synovial tissues of mice with different treatments. n = 3, mean ± SD. Significant differences were determined via one-way ANOVA analysis with Tukey’s multiple comparison test. **p* <0.05, ***p* <0.01, ****p* <0.001, *****p* <0.0001, ns stands for no significant difference.

**Figure S23.** Representative images and corresponding quantification of extracellular pH (medium pH) of cells in the indicated groups. n = 3, mean ± SD. Significant differences were determined via Student’s *t* test. ****p* <0.001.

**Figure S24.** FCM profiles and corresponding quantification of the intensity Cy7-LAC within RAW264.7 cells at different time points. n = 3, mean ± SD. The significant differences are determined by one-way ANOVA analysis with Tukey’s multiple comparison test. ***p* < 0.01, *****p* < 0.0001. ns stands for no significant difference.

**Figure S25.** Western blots (A) and corresponding quantification of the expression of CD206 (B), iNOS (C), and COX-2 (D) in cells treated with LAC in gradient concentrations. CD206 and iNOS were normalized to β-Actin, and COX-2 was normalized to GAPDH. n = 3, mean ± SD. The significant differences are determined by one-way ANOVA analysis with Tukey’s multiple comparison test. **p* <0.05, ***p* <0.01, ****p* <0.001, ns stands for no significant difference.

**Figure S26.** Semi-quantification of the expression of iNOS (A), CD206 (B), IRF5 (C), COX-2 (D), and the phosphorylation of STAT6 (E) in cells with different treatments. n = 3, mean ± SD. The significant differences are determined by one-way ANOVA analysis with Tukey’s multiple comparison test. **p* <0.05, ***p* <0.01, ****p* <0.001, *****p* <0.0001, ns stands for no significant difference.

**Figure S27.** Representative western blots and corresponding quantification of the expression of CD206 and iNOS in THP-1 cells with different treatments. n = 3, mean ± SD. The significant differences are determined by one-way ANOVA analysis with Tukey’s multiple comparison test. **p* < 0.05, ***p* < 0.01, ****p* < 0.001, *****p* < 0.0001, ns stands for no significant difference.

**Figure S28.** FCM profiles (A) and corresponding quantification of M2 macrophages in the indicated groups. n = 3, mean ± SD. The significant differences are determined by one-way ANOVA analysis with Tukey’s multiple comparison test. ****p* < 0.001, *****p* < 0.0001, ns stands for no significant difference.

**Figure S29.** Semi-quantification of the fluorescence intensity of iNOS in cells with different treatments. n = 3, mean ± SD. The significant differences are determined by one-way ANOVA analysis with Tukey’s multiple comparison test. **p* < 0.05, ***p* < 0.01, *****p* < 0.0001, ns stands for no significant difference.

**Figure S30.** Representative western blots (A) and corresponding quantification of the expression of CD206 (B), IRF5 (C), and the phosphorylation of STAT6 (D) in the indicated groups. n = 3, mean ± SD. The significant differences are determined by one-way ANOVA analysis with Tukey’s multiple comparison test. ns stands for no significant difference.

**Figure S31.** Western blots (A) and corresponding quantification of the expression of netrin-1 in cells with different treatments (B). n = 3, mean ± SD. The significant differences are determined by one-way ANOVA analysis with Tukey’s multiple comparison test. **p* < 0.05, ***p* < 0.01, ns stands for no significant difference.

**Figure S32.** Quantification of the TRAP-positive multinucleated cells (MNCs) in the indicated groups. n = 3, mean ± SD. The significant differences are determined by one-way ANOVA analysis with Tukey’s multiple comparison test. ***p* < 0.01, *****p* < 0.0001.

**Figure S33.** Schematic illustration of the macrophage and DRG explant co-culture system.

**Figure S34.** Representative western blots of the expression of NGF in cells with different treatments.

**Figure S35.** Quantification of the relative density of TRPV^+^ cells in the knee joints of mice with different treatments. n = 3, mean ± SD. The significant differences are determined by one-way ANOVA analysis with Tukey’s multiple comparison test. **p* < 0.05, *****p* < 0.0001, ns stands for no significant difference.

**Figure S36.** Representative IF images (A) and corresponding quantification of the MFI of CGRP in the knee joints of mice with different treatments. n = 3, mean ± SD. The significant differences are determined by one-way ANOVA analysis with Tukey’s multiple comparison test. **p* < 0.05, *****p* < 0.0001, ns stands for no significant difference.

**Figure S37.** Representative micro-CT images (A) and corresponding measurements of BV/TV (B), BS/TV (C), Tb.Pf (D), Tb.Th (E), Tb.N (F), Tb.Sp (G), and BS/BV (H). n = 3, mean ± SD. The significant differences are determined by one-way ANOVA analysis with Tukey’s multiple comparison test. ***p* < 0.01, ns stands for no significant difference.

**Figure S38.** Quantification of the MFI of iNOS in synovium of mice with different treatments. n = 3, mean ± SD. The significant differences are determined by one-way ANOVA analysis with Tukey’s multiple comparison test. **p* < 0.05, *****p* < 0.0001, ns stands for no significant difference.

**Figure S39.** Quantification of the relative density of TRAP^+^ cells in the knee joints of mice with different treatments. n = 3, mean ± SD. The significant differences are determined by one-way ANOVA analysis with Tukey’s multiple comparison test. **p* < 0.05, ***p* < 0.01, ns stands for no significant difference.

**Figure S40.** The osteophyte number (A) and volume of calcified meniscus and synovial tissues (B) in the knee joints of mice with different treatments. n = 6, mean ± SD. The significant differences are determined by one-way ANOVA analysis with Tukey’s multiple comparison test. ***p* < 0.01, ****p* < 0.001, *****p* < 0.0001.

**Figure S41.** Biocompatibility of LAC. (A) Establishment and therapeutic regimen of the DMM mouse model used for biocompatibility assessments. (B) H&E staining of major organs from DMM mice after different treatments. (C–E) Serum biochemistry measurements of ALT (C), AST (D), and BUN (E) levels. (F-I) complete cell count tests. n = 3, mean ± SD. Significant differences were determined via one-way ANOVA analysis with Tukey’s multiple comparison test. ns stands for no significant difference.

**Figure S42.** Serum biochemistry measurement of creatinine. n = 3, mean ± SD. The significant differences are determined by one-way ANOVA analysis with Tukey’s multiple comparison test. ns stands for no significant difference.

**Figure S43.** Complete blood counts tests. n = 3, mean ± SD. The significant differences are determined by one-way ANOVA analysis with Tukey’s multiple comparison test. ns stands for no significant difference.
